# Viruses and Their Interactions With Bacteria and Archaea of Hypersaline Great Salt Lake

**DOI:** 10.3389/fmicb.2021.701414

**Published:** 2021-09-28

**Authors:** Bishav Bhattarai, Ananda S. Bhattacharjee, Felipe H. Coutinho, Ramesh K. Goel

**Affiliations:** ^1^Department of Civil and Environmental Engineering, The University of Utah, Salt Lake City, UT, United States; ^2^Carl R. Woese Institute for Genomic Biology, The University of Illinois at Urbana-Champaign, Urbana, IL, United States; ^3^Departamento de Producción Vegetal y Microbiología, Universidad Miguel Hernández, Alicante, Spain

**Keywords:** viral diversity, virus–host association, hypersaline lake, lysogens, auxiliary metabolic genes

## Abstract

Viruses play vital biogeochemical and ecological roles by (a) expressing auxiliary metabolic genes during infection, (b) enhancing the lateral transfer of host genes, and (c) inducing host mortality. Even in harsh and extreme environments, viruses are major players in carbon and nutrient recycling from organic matter. However, there is much that we do not yet understand about viruses and the processes mediated by them in the extreme environments such as hypersaline habitats. The Great Salt Lake (GSL) in Utah, United States is a hypersaline ecosystem where the biogeochemical role of viruses is poorly understood. This study elucidates the diversity of viruses and describes virus–host interactions in GSL sediments along a salinity gradient. The GSL sediment virosphere consisted of *Haloviruses* (32.07 ± 19.33%) and members of families *Siphoviridae* (39.12 ± 19.8%), *Myoviridae* (13.7 ± 6.6%), and *Podoviridae* (5.43 ± 0.64%). Our results demonstrate that salinity alongside the concentration of organic carbon and inorganic nutrients (nitrogen and phosphorus) governs the viral, bacteria, and archaeal diversity in this habitat. Computational host predictions for the GSL viruses revealed a wide host range with a dominance of viruses that infect *Proteobacteria*, *Actinobacteria*, and *Firmicutes*. Identification of auxiliary metabolic genes for photosynthesis (*psbA*), carbon fixation (*rbc*L, *cbb*L), formaldehyde assimilation (SHMT), and nitric oxide reduction (*Nor*Q) shed light on the roles played by GSL viruses in biogeochemical cycles of global relevance.

## Introduction

Viruses are the most abundant biological entities (Bergh et al., 1989; Fuhrman, 1999; Suttle, 2005; Nooij et al., 2018; Graham et al., 2019) which infect all forms of known cellular life. Viruses of bacteria and archaea act as a repository of genetic information that influences the evolution and ecophysiology of their host. Each viral infection event can introduce new genetic information into the host genome (Suttle, 2005). Viral infections are a major source of host mortality altering microbial community composition and thus affecting the ecosystems fluxes of nutrients and energy (Weinbauer and Rassoulzadegan, 2004; Rodriguez-Brito et al., 2010; Cram et al., 2016). Viruses hijack cellular machinery of their hosts for viral replication and propagation, while reprogramming the host’s cellular metabolism (Forterre, 2013; Rosenwasser et al., 2016). Viruses can act as agents for the horizontal transfer of ecologically important genes (Jiang and Paul, 1998). Viruses also encode AMGs, host derived genes that are expressed during infection to re-direct hosts metabolism toward pathways that promote viral productivity (Sullivan et al., 2006; Hurwitz and U’Ren, 2016). These genes, AMG, are involved in various metabolisms, including sulfur, nitrogen, and methane, in DNA replication, repair, recombination, and amino acid biosynthesis, and are also detected in extreme environments (Anantharaman et al., 2014; Anderson et al., 2014; Ahlgren et al., 2019). AMGs interfering with carbon fixation and methylotrophy metabolism have been detected in viruses from the lake Baikal (Coutinho et al., 2020). AMGs encoding photosynthetic reaction centers in cyanophages highlight their potential to boost photosynthetic metabolism (Sullivan et al., 2006; Lindell et al., 2007). Cyanophages from the marine environment have also been observed with genes for CP12 (Thompson et al., 2011). The expression of CP12 in the cyanophages’ host causes the switching of carbon flux from the Calvin cycle to the pentose phosphate pathway (PPP). Such AMGs exemplify how viruses can affect biogeochemical cycles of global relevance in ways which beyond killing of their hosts.

Saline lakes, which globally display similar volume (1.04 × 10^5^ km^3^) as freshwater lakes (1.25 × 10^5^ km^3^) (Horne and Goldman, 1994), are traditionally deemed of little significance and understudied as they are not easily monetized (Wurtsbaugh et al., 2017). Nevertheless, these saline habitats are critical, have critical roles in the regulation of climate, and global geochemistry (Hoehler et al., 2001; Daffonchio et al., 2006; Sternai et al., 2017). The GSL in the United States represents such an ecosystem. The GSL is the largest terminal saline lake in the Western Hemisphere (Keck and Hassibe, 1979). Between 1955 and 1959, a railway causeway was constructed on the GSL, dividing the lake into north and south arm (Madison, 1970; Cannon and Cannon, 2002; Baxter et al., 2005). The north arm of GSL receives no to little freshwater and displays salinity levels between 28 and 34%. The south arm is fed by different tributaries and displays salinity between 11 and 15% (Baxter et al., 2005; Almeida-Dalmet et al., 2015). Terminal lakes without any water discharge outlet have a higher degree of susceptibility to climate change with varying cycles of drought and flooding (Wurtsbaugh et al., 2017). In the GSL, the rise of lake water level, as high as four meters, between 1983 and 1987 was observed, followed by drought events resulting in recorded lowest elevation (since 1963) recorded in 2016 (Stephens, 1990). The fluctuations in GSL water levels lead to salinity changes that affect the resident microbial community (Wu et al., 2006; Herlemann et al., 2011; Logares et al., 2013).

The microbial community in the northern arm of the GSL has been shown to be less impacted by fluctuations in temperature and salinity (Almeida-Dalmet et al., 2015). In contrast, the lesser saline southern arm has been shown to harbor a diverse and dynamic microbial community as it receives fresh streamflow from the Bear, Weber, and Jordan rivers (Meuser et al., 2013; Boyd et al., 2014; Lindsay et al., 2017; Baxter and Butler, 2020). Although characterization of microbial community and their metabolism in the GSL has advanced, diversity of viruses and their ecological significance in the southern arm of the GSL has still not been explored in detail. Viral studies in the GSL are limited to identifying *Haloviruses* and their role in infecting halophilic bacteria and archaea (Post, 1981; Baxter et al., 2011; Shen et al., 2012). Recently, Motlagh et al. (2017) provided an insight into GSL sediment virosphere, which was shown to encode genes for nitrogen, carbon, and sulfur cycling (Motlagh et al., 2017). However, the impact of salinity on the viral community and their interaction with the host community of bacteria and archaea remains underexplored. Furthermore, little is known about the diversity of AMGs in hypersaline ecosystems and its potential impacts in biogeochemical cycles of global relevance.

Here, we delineate the bacterial, archaeal, and viral diversity of GSL sediments. To our knowledge, this is the first study of the viral community along the north-south salinity gradients in the GSL. We hypothesize that across the GSL, the diversity of bacteria, archaea, and viruses reduces with an increase in salinity. The objectives of our study were to (a) delineate the viral diversity of GSL, an extreme environment, and (b) elucidate the role of hypersaline viruses in nutrient cycling. Viral, bacterial, and archaeal metagenomes were obtained from three GSL sediments sites along the lake’s north-south transect. From these samples we recovered and characterized viral, bacterial, and archaeal draft genomes for the genome-based ecology study. Viruses were assigned to their putative bacterial and archaeal hosts through computational approaches. In addition, AMGs were identified among the viruses from GSL and their potential ecological roles were characterized.

## Materials and Methods

### Sample Collection and Measurement of Sediment and Water Quality Parameters

**FIGURE 1:**
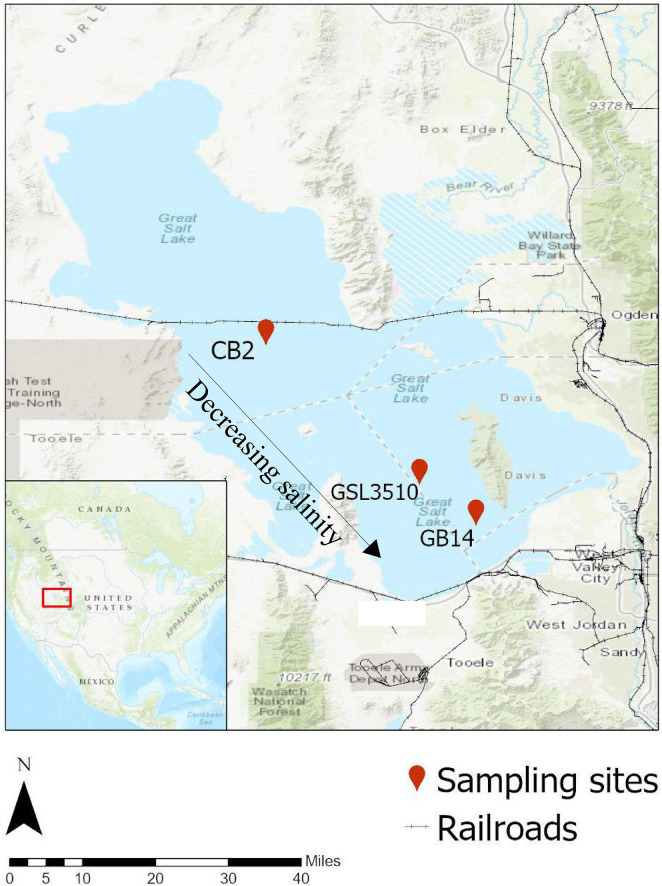
Location of study areas in the Great Salt Lake (GSL). The three sites are (i) CB-2, 12.3 miles North-West of Carrington Island (41°10′32.3″ N, 112°39′11.9″ W); (ii) GSL 3510, 6 miles West of Antelope Island (40°53′56.0″ N, 112°20′56.0″ W); and (iii) GB-14, 6.8 miles North of Great Salt Lake Marina (40°49′06.2″ N, 112°14′13.6″ W). Source: Background Map (Topographic Base Map, ESRI 2020).

Three sampling sites were chosen in Gilbert Bay and Carrington Bay (southern arm) of GSL on the north to south transect covering a salinity gradient (Figure 1). The three sites on the GSL were (i) CB2, 12.3 miles North-West of Carrington Island (41°10′32.3″ N, 112°39′11.9″ W); (ii) GSL 3510, 6 miles west of Antelope Island (40°53′56″ N, 112°20′56″ W); and (iii) GB-14, 6.8 miles north of Great Salt Lake Marina (40°49′06.2″ N, 112°14′13.6″ W). The samples were collected on 10th August 2017. The flow of high-density brine from the northern arm to the southern arm through the causeway fill material and culverts causes denser brine to settle in the deepest section, thus creating a deep brine layer in the GSL. The deep brine water sample was collected ∼0.5 m from the bottom of the GSL using a peristaltic pump following the United States Geological Survey’s (USGS) and GSL monitoring program water sampling standard operating procedure (SOP) in a 1L HDPE sampling bottle (Wilde, 2011). The tube was cleaned with dilute hydrochloric acid and deionized water three times to remove any previous sampling carryover. Sediment samples were collected from the surface of the GSL sediments with a stainless-steel box corer (Wildco, FL, United States) as performed in Motlagh et al. (2017). The collected samples were immediately placed inside a storage box with ice and shipped to the laboratory within 4 h of sample collection.

Temperature, pH, specific conductivity, ORP, depth, and DO were measured using an onsite sounder equipped with various sensors (YSI 600 XL). The sediment samples were centrifuged at 2,000 × *g* for 10 min to extract the interstitial water as pore water. The surface water and the extracted pore water were filtered through 0.45 μm mixed cellulose hydrophilic filter paper before further analyses (Millipore, MA, United States). The concentration of ammonium (NH_4_-N), nitrite (NO_2_-N), nitrate (NO_3_-N), orthophosphate (PO_4_-P), and TOC were measured for both the surface and pore water. Ammonium (NH_4_-N) and TOC were measured using the HACH methods TNT830 and HACH 10128, respectively, according to the manufacturer’s instructions (HACH, CO, United States). Nitrite (NO_2_-N), nitrate (NO_3_-N), and orthophosphate (PO_4_-P) were analyzed using Ion Chromatography (IC) (Metrohm 883 Basic IC plus) (Pfaff, 1993).

### Preparation of Viral Concentrates and Viral Nucleic Acid Extraction

The viral concentrates were prepared according to the protocol used by Motlagh et al. (2017) for hypersaline sediments. For each site, 300 g of sediments were mixed with three times the volume of autoclaved and filtered 1% (w/v) potassium citrate buffer (10 g/l potassium citrate, 1.44 g/l of disodium phosphate, 0.24 g/l of monopotassium phosphate, pH 7). The mixture was placed on a shaker at 160 rpm on ice overnight to detach the free viruses from the sediment mass and bring them into the solution. Following the overnight shaking, the sample mixture was centrifuged at 7800 × *g* at 4°C on an Avanti J-E centrifuge (Beckman Coulter, CA, United States) in J-LITE^®^JLA-10.500 Fixed-Angle Aluminum Rotor for 45 min to pellet down the bacterial debris as well as the sediments. The retained supernatant was then sequentially filtered through various pore-sized membrane filter units (mixed cellulose ester filters; 1.2 μm, 0.8 μm, 0.45 μm, 0.22 μm) to avoid any contamination from microbial cells. The filtrate was centrifuged overnight at 12,300 × *g* at 4°C to pellet the virus particles. The pellet was re-suspended in SMG buffer (5.8 g/l sodium chloride, 2 g/l magnesium sulfate, 5 ml/l of 5% (w/v) gelatin, 50 ml/l of 1M Tris-Cl, pH 7.5) and filtered through 0.22 μm pore size filter paper (Millipore Co., MA, United States) to remove any residual bacterial cell and sediment debris.

The viral particles in the SMG buffer were purified using cesium chloride (CsCl) density gradient at 1.4–1.6 g/cc density by isopycnic centrifugation at 35,000 rpm, 20°C for 3 h (Angly et al., 2006). The cesium chloride purification was carried out twice to ensure the highest removal of any residual bacterial cell debris and other particles, thereby guaranteeing extracted viral particles’ purity.

The purified virus samples were dialyzed overnight through dialysis tubing with a nominal MWCO of 3500 at 4°C against SMG buffer. 10 μL of the CsCl purified virus samples were loaded on Formvar/Carbon 400 mesh grids and allowed to settle for a minute. The sample was blotted out using bibulous paper, and the grids were negatively stained with 1% (w/v) uranyl acetate for 1 min. Excess staining liquid was removed using a bibulous paper. Before examining under JEOL JEM-1400 TEM (Tokyo, Japan), the grid was air-dried at an accelerating voltage of 120 kV.

DNA was extracted from purified viral particles based on a spin column purification method using a phage DNA isolation kit (Norgen Biotek Corp., Canada). The viral sample was subjected to DNase treatment with RNase-free DNase I (Thermo Scientific, CA, United States) at 37°C for 30 min, followed by the DNase I inactivation at 75°C for 5 min before the lysis process to remove any residual microbial DNA and digest any free DNase in the sample. Since it is difficult to obtain a high yield of viral DNA from environmental samples, 4 μl of Proteinase K (20 mg/mL) (Promega, Madison, WI, United States) was added to the sample incubated at 55°C for 30 min to increase viral DNA yield. The extracted DNA’s quantity and quality were checked on a NanoDrop ND 2,000 spectrophotometer (Thermo Scientific, United States) at 260 and 280 nm. PCR amplification of the hypervariable V4-V9 region was performed using 515/1492R universal 16S rRNA gene primer set as a test to confirm the absence of microbial DNA contamination in the viral DNA extract (Diemer and Stedman, 2012). The PCR products were electrophoresed in a 1% agarose gel stained with ethidium bromide (10 μg/ml) and visualized under UV illumination (Molecular Imager Gel Doc XR+, BIO-RAD).

### Genomic DNA Extraction From Microbes

The genomic DNA was extracted from 0.5 g of sediment samples according to the manufacturer’s instructions using DNeasy PowerSoil Kit (QIAGEN). For each site. the genomic DNA was extracted in triplicates and later pooled together. Similar to viral nucleic acid, microbial genomic DNA concentration was measured using NanoDrop ND 2,000 spectrophotometer (Thermo Scientific, United States).

### DNA Library Preparation and Sequencing

For both the microbial and viral DNA samples, library construction was performed using the Swift Biosciences Accel-NGS 1S Plus DNA Library Kit. Briefly, around 50 ng of genomic DNA was heat-denatured and hybridized with oligonucleotides consisting of random hexamers linked to Illumina P5 adapter sequences. Strand replication was accomplished using EpiGnome polymerase. Double-stranded DNA was heat-denatured to enable ligation of the EpiGnome Terminal Tagging Oligo, which adds Illumina P7 adapter sequence to the 3′ end of the replicated strand. Adapter-ligated DNA molecules were enriched by ten cycles of PCR, and the amplified library was subsequently purified using Agencourt AMPure XP beads (Beckman Coulter Genomics, CA, United States). The library’s concentration was measured using the Qubit dsDNA HS Assay (Invitrogen, CA, United States). An aliquot of the library was resolved on an Agilent 2200 Tape Station using a D1000 assay to define the sequencing library’s size distribution. Libraries were adjusted to a concentration of approximately 10 nM, and quantitative PCR was performed using the Kapa Library Quant Kit (Kapa Biosystems, MA, United States) to calculate the molarity of adapter-ligated DNA molecules. The concentration was further adjusted following qPCR to prepare the library for Illumina sequence analysis. The samples were sequenced on an Illumina MiSeq Benchtop DNA sequencer (Illumina, CA, United States) with 300-cycles paired-end at Core Facility, HCI, University of Utah. This study attempts to avoid applying any amplification process to minimize biases for the virus metagenomics and its analysis.

### Metagenome Sequence Processing and Analysis

Quality control, assembly, and gene prediction for both metagenome and metavirome datasets were performed simultaneously using the same pipeline. The raw reads generated by the Illumina MiSeq sequencer were first checked for quality using FastQC v0.11.8 (Andrews, 2010) and then trimmed using Trimmomatic v0.38 (Bolger et al., 2014). The read-through adapters (ILLUMINACLIP: Swift-PE.fa:2:30:10), low-quality base calls at the start and end of reads (LEADING:3, TRAILING:3), reads with an average Phred score lower than 20 in a sliding window of 4 bp (SLIDINGWINDOW:4:20) were trimmed from the raw reads. Also, sequencing reads less than 100 bp were discarded from further analyses (MINLEN:100).

The quality-controlled reads were *de novo* assembled using metaSPAdes v3.13.0 (Bankevich et al., 2012) with a range of kmer values (21, 33, 55, 77, 99, 127), and the best-assembled scaffolds, as reported by SPAdes, were chosen. Only the contigs longer than 1 kbp were retained and referred to as ‘RMC’ and ‘RVC’ for the microbial and viral dataset, respectively (Supplementary Figure S2).

### Taxonomic Classification and Open Reading Frame Prediction of Viral, Bacterial, and Archaeal Sequences

Viral contigs were identified in the RMC and RVC using VirSorter (Roux et al., 2015). “Viromes” database- a combination of RefSeqABVir sequences and virome sequences sampled from freshwater, seawater, and human gut- was used as a reference database allowing for the detection of new viruses (Roux et al., 2015). For each of the three sites, viral contigs identified within the RMC and RVC were grouped and named as ‘CVC’ from here onward. Similarly, ‘CMC’ consisted of RMC with viral contigs removed. Supplementary Figure S2 shows the schematic workflow of the abovementioned procedure. The relative percentage abundance of CVCs was obtained by mapping the reads from both metagenome and metavirome datasets for individual sites. NCBI RefSeq Viral database and Integrated Microbial Genome/Virus (IMG/VR) database (Paez-Espino et al., 2019) filtered for viral genomes identified in freshwater, marine, saline, and hypersaline environments were used for taxonomic annotation of viral genomes. The RefSeq Viral database and the filtered IMG/VR database are referred to as RVG and EVG databases. The viral contigs were annotated for taxonomic classification against the RVG and EVG using tblastx v.2.9.0 (e-value 10^–5^) and the best blast hit was selected using enveomics (Rodriguez-R and Konstantinidis, 2016). The lifestyle of the viruses based on the conserved protein domains were predicted using BACPHLIP (Hockenberry and Wilke, 2021). The bacterial and archaeal communities were profiled using Metaphlan version 2.7.7 (Truong et al., 2015) using default parameters. ORFs were identified in the CMC and CVC using Prodigal v2.6.3 (Hyatt et al., 2010). ORFs were annotated using SUPER-FOCUS (e-value 10^–5^) (Silva et al., 2016). The functional profile was constructed by mapping the trimmed reads to the predicted ORFs using BBMap (Bushnell, 2014).

### Binning of Bacterial and Archaeal Contigs and Phylogenetic Analysis

The RMCs were binned into draft bacterial and archaeal genomes. First, the mean coverage of contigs was calculated by mapping the quality-controlled reads to the RMCs using Bowtie2 (Langmead and Salzberg, 2012). Then, using default parameters in MetaBAT2 (Kang et al., 2019), microbial and archaeal contigs were binned into draft metagenome-assembled genomes (MAGs). The recovered MAGs were profiled and checked for quality (genome completeness and contamination) using CheckM v1.0.7 (Parks et al., 2015). MAGs with contamination greater than 5% were manually refined in Anvi’o using differential coverage, tetranucleotide frequency, and marker gene content (Eren et al., 2015). Post refinement of the MAGs, the phylogenetic markers, and relative abundance of the MAGs was assessed using ‘tree_qa’ and ‘profile’ commands, respectively, in CheckM (Parks et al., 2015). The taxonomy of the MAGs was also determined using the Genome Taxonomy Database Toolkit (GTDB-Tk) (Chaumeil et al., 2019). The MAGs with contamination less than 5% were placed in the reference tree inferred from the concatenation of 43 conserved marker genes using CheckM v1.0.7 (Parks et al., 2015). The reference tree was annotated to distinguish different ‘Class’ levels and identify the MAGs from different sites in the GSL using CLC Genomics Workbench v.12 (CLC Bio, Denmark). The branches of the reference tree represent ‘Class’ taxonomy. Each ‘Class’ was denoted with individual color. The branches with the GSL MAGs were expanded to show their taxonomic position in the phylogenetic tree. The MAGs from individual sites in the GSL are distinguished by different symbols. The MAGs were annotated using Prokka v1.14.6 (e-value 10^–5^) (Seemann, 2014). The annotated proteins for selected MAGs were parsed through MicrobeAnnotator (Ruiz-Perez et al., 2020) to check for the genome’s metabolic potential based on KEGG modules.

### Computational Host Prediction of Viral Sequences

From the CMCs, clustered regularly interspaced short palindromic repeats (CRISPR) arrays were predicted with CASC, the CRISPR detection and validation tool (Nasko et al., 2019). The number of unique and shared spacers was determined by clustering the ‘bonafide’ spacer sequences at 97% nucleotide similarity using CD-HIT-EST (Li and Godzik, 2006). The correct spacers were used as query sequences to search against the CVCs, RVG, and EVG using Blastn (word size 7). The blastn hits were filtered using specific parameters (minimum alignment length: 15 bp; minimum percentage identity: 90%; minimum query coverage: 95%; evalue ≤ 0.1; maximum one nucleotide mismatch) to be considered a significant hit. Bacterial and archaeal contigs were annotated against the NCBI non-redundant nucleotide database using tblastx v.2.9.0.

Viruses were also assigned to their putative hosts by comparing the shared genetic content between viruses and hosts described in Coutinho et al. (2020). Bacterial and Archaeal genomes from the NCBI RefSeq database were used as reference genomes. GTDB was used to assign taxonomy to the reference genomes. CRISPR spacers (three points), homology matches (two points), and shared tRNAs (one point) were analyzed as virus–host association signals as described in Coutinho et al. (2020).

### Data Analysis

The data analysis and visualizations in this study were conducted using Microsoft Excel, Python 3, and Origin 2020b. The phylogenetic tree was produced using CLC Genomics Workbench (CLC Bio, Denmark).

## Results

### Water Quality and Sediment Characteristics

Water chemistry parameters (temperature, pH, specific conductivity, ORP, DO) were measured onsite in three sampling locations at the Great Salt Lake (Table 1 and Supplementary Figure S1). The surface and sediment pore-water’s water quality parameters (inorganic nitrogen, phosphorus content, and organic carbon) were measured and summarized in Table 1. Salinity at sites CB2, GSL3510, and GB14 (Figure 1) was measured to be 17.59% (175.9 ppt), 12.14% (121.4 ppt), and 11.66% (116.6 ppt), respectively, demonstrating salinity differences across the sampled sites. Further, variability in DO in the deep layer of the overlying water column was observed with the oxygen-rich zone in GB14 (DO: 6.84 mg/L), oxygen deficiency in GSL 3510 (DO: 0.44 mg/L), and anoxia in CB2 (DO ∼ 0 mg/L) (Table 1). In terms of carbon and nutrients availability, sediments from site CB2 consisted of a higher concentration of TOC, nitrogen (N), and phosphorus (P) than GSL3510 and GB14 sites (Table 1). The extracted sediment pore water had higher TOC, ammonical-nitrogen (NH_4_-N), and phosphate-phosphorous (PO_4_-P) concentration than the overlying water column at all GSL sites (Table 1).

**TABLE 1:** Environmental properties determined for sediments, pore water (included within parentheses), and lake water sampled on 10th August 2017 from three locations in the Great Salt Lake.

Parameters	CB2	GSL 3510	GB14
Salinity (ppt)	175.9	121.4	116.6
Specific conductivity (μS/cm)	200800	151700	147000
Oxidation–reduction potential (mV)	−358	−304	−81
Dissolved oxygen (mg/L)	0	0.44	6.84
Dissolved oxygen saturation (%)	0	12.7	196.5
pH	7.38	7.83	8.19
Total organic carbon (mg/L)	93.15 ± 0.81 (126.21 ± 0.37)	59.12 ± 0.40 (90.44 ± 0.47)	60.09 ± 0.64 (89.72 ± 0.64)
NH_3_-N (mg/L)	0.66 ± 0.06 (34.33 ± 0.42)	0.06 ± 0.02 (8.54 ± 0.09)	0.01 ± 0.00 (7.06 ± 0.11)
NO_2_-N (mg/L)	0.03 ± 0.00 (0.11 ± 0.01)	0.01 ± 0.00 (0.04 ± 0.01)	0.01 ± 0.00 (0.07 ± 0.00)
NO_3_-N (mg/L)	1.09 ± 0.02 (0.99 ± 0.03)	0.6 ± 0.06 (0.77 ± 0.02)	0.65 ± 0.02 (0.7 ± 0.01)
PO_4_-P (mg/L)	0.16 ± 0.00 (4.71 ± 0.05)	0.11 ± 0.00 (1.22 ± 0.02)	0.12 ± 0.00 (1.51 ± 0.02)

### Morphological Diversity of Viruses in Great Salt Lake Sediments

**FIGURE 2:**
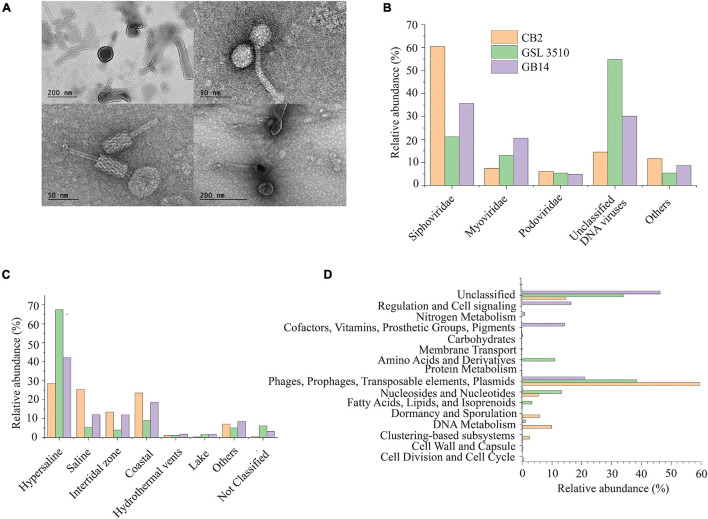
**(A)** Morphological diversity of the viruses in the Great Salt Lake sediments observed via the transmission electron microscope (TEM). **(B)** Family level viral community composition from the Great Salt Lake sediments. **(C)** Ecosystem-based community composition of viruses from the Great Salt Lake sediments based on the similarity with viruses identified in different aquatic ecosystems (IMG/VR database). **(D)** Functional classification (SEED subsystem Level I) of the viral open reading frames (ORFs).

The purified viral particles observed under a TEM show tailed and non-tailed viral morphologies of the GSL sediments (Figure 2A). The tailed morphologies belong to the order *Caudovirales*, tailed viruses that infect bacteria and archaea, which are prevalent in hypersaline environments (Sencilo and Roine, 2014). Spherical, head-tail, and lemon-shaped viral morphologies were also observed. The morphological characterization, however, cannot establish the taxonomic identity of the viruses. Therefore, genomic analysis was incorporated to elucidate the viral diversity of hypersaline GSL.

### Bioinformatic Analysis of Bacterial, Archaeal, and Viral Metagenomes

Following the quality trimming, an average of 95.92% metagenomic reads and 96.87% metavirome reads were retained (Supplementary Table S1). The assembly statistics and the number of ORFs detected from each site is shown in Supplementary Table S2.

From both metagenomes and metaviromes assembly, VirSorter (Roux et al., 2015) identified a total of 3,295 contigs (∼1 to 74.42 kbp) as viral and 21 contigs as prophages (∼9.68 to 1784.23 kbp). The number of VirSorter (Roux et al., 2015) identified viruses and prophages from both the metagenomes and metaviromes dataset in each site is described in Supplementary Table S3. The contigs identified as viral contigs by VirSorter were referred to as viral genomes. A total of 578, 81, and 41 viral contigs from CB2, GSL3510, and GB14 had sizes greater than 5 kbp.

### Taxonomic and Functional Diversity of Bacterial and Archaeal Community Along the Salinity Gradient in the Great Salt Lake Sediments

**FIGURE 3:**
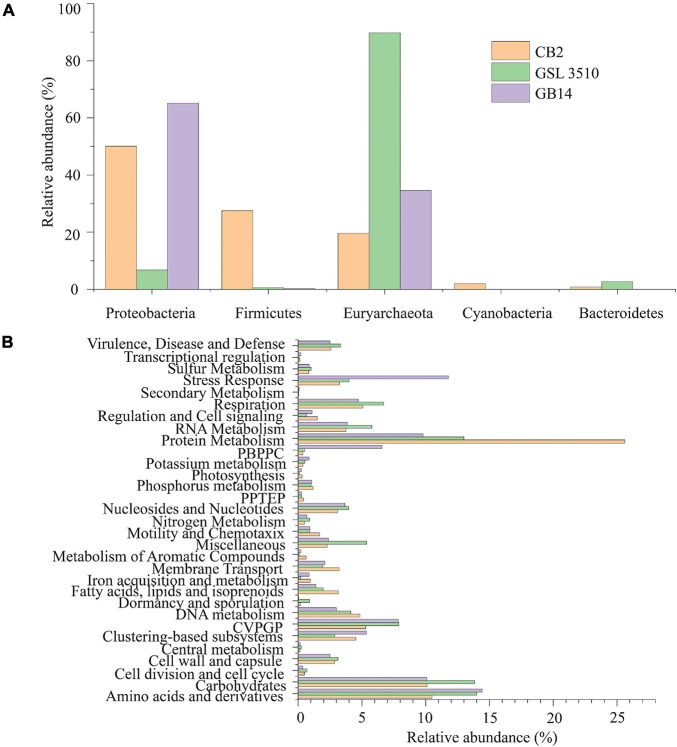
**(A)** Phylum level classification of the bacterial and archaeal community across the Great Salt Lake sediments. **(B)** Functional classification (SEED subsystem Level I) of the bacterial and archaeal open reading frames (ORFs). (PPTEP, phages, prophages, transposable elements, plasmids; CVPGP, cofactors, vitamins, prosthetic groups, pigments; PBPPC, predictions based on plant-prokaryote comparative analysis).

The taxonomic profiling with MetaPhlAn v2.7.7 (Truong et al., 2015) revealed that 19.60, 89.74, and 34.56% of the quality filtered reads were respectively assigned to archaea in samples CB2, GSL3510, and GB14 while remaining ones were assigned bacteria. More than half of the quality-filtered reads were taxonomically classified to be *Euryarchaeota*, followed by *Proteobacteria* in sediment samples across three sites in GSL (Figure 3A). Bacterial and archaeal sequences belonging to genus *Thioalkalivibrio*, *Halorubrum*, *Methanohalophilus*, *Halobacterium*, and family *Halanaerobiaceae* were identified at the three GSL sites (Supplementary Figures S4D,E). Archaea belonging to the *Haloarcula* genus was found to be in sediments of CB2 and GSL 3510. Genus *Halanaerobium*, *Marinobacter*, *Halomonas*, *Haloquadratum*, and *Nodularia*, which were not found in GSL 3510 and GB14, were identified in CB2. Similarly, the genus *Gillisiae* was only identified at site GSL 3510. The Shannon diversity index of the bacterial and archaeal community across the GSL was calculated at the species level. The Shannon diversity index (H) of site CB2 was the highest (*H* = 0.89), followed by GSL 3510 (*H* = 0.74) and GB14 (*H* = 0.42). In the GSL, bacterial and archaeal diversity represented by Shannon index (*H*) is in congruence with decreasing salinity. This is contrasting as hypersaline environments are constrained in bacterial and archaeal diversity due to extreme environmental conditions; an increase in salinity leads to decreased bacterial and archaeal diversity (Ventosa et al., 2014; Ji et al., 2019).

The predicted genes of the contigs were queried against the SEED subsystem database to predict the community’s metabolic potential. The relative abundances of the microbial sequences that had similarities to the SEED subsystem categories are depicted in Figure 3B. In GSL, a maximum number of predicted genes were affiliated to protein metabolism, SEED system category (Figure 3B). Amino acids and derivatives and carbohydrates comprised 12.97 ± 2.15 and 11.34 ± 2.15% (mean ± SD), respectively, of the genes. Genes affiliated to stress response were also identified among the contigs. Interestingly, 11.78% of predicted ORFs of GB14 (salinity 11.66%) were affiliated to stress response, greater than the other two study areas (Figure 3B). The bacterial and archaeal stress factors may not be solely attributed to salinity gradients. Gene for sulfur and nitrogen metabolism were identified for the bacteria and archaea involved in the GSL biogeochemical cycles.

### Bacterial and Archaeal Metagenome-Assembled Genomes of the Great Salt Lake

**FIGURE 4:**
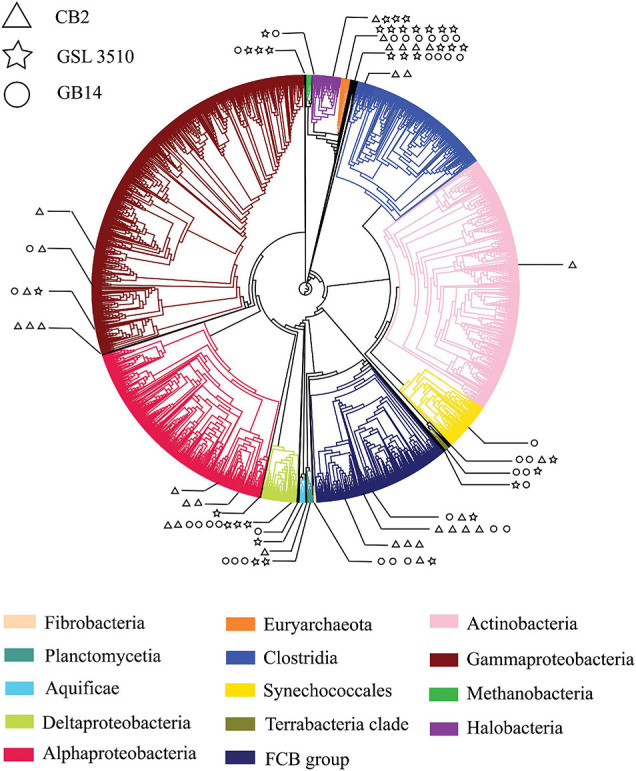
A phylogenetic tree of Great Salt Lake metagenome-assembled genomes (MAGs). The MAGs with contamination (>5%) assessed by CheckM v1.0.7 (Parks et al., 2015) were placed in the reference phylogenetic tree inferred with 43 conserved marker genes using CheckM v1.0.7 (Parks et al., 2015). The phylogenetic tree was annotated with CLC Genomics Workbench v.12 (CLC Bio, Denmark). The branch of the reference tree represents ‘Class,’ taxonomy. Each ‘Class’ is denoted with individual color. The branches with Great Salt Lake MAGs are expanded to show their taxonomic position on the phylogenetic tree. The Great Salt Lake MAGs from three sites on the Lake are distinguished with different symbols.

**FIGURE 5:**
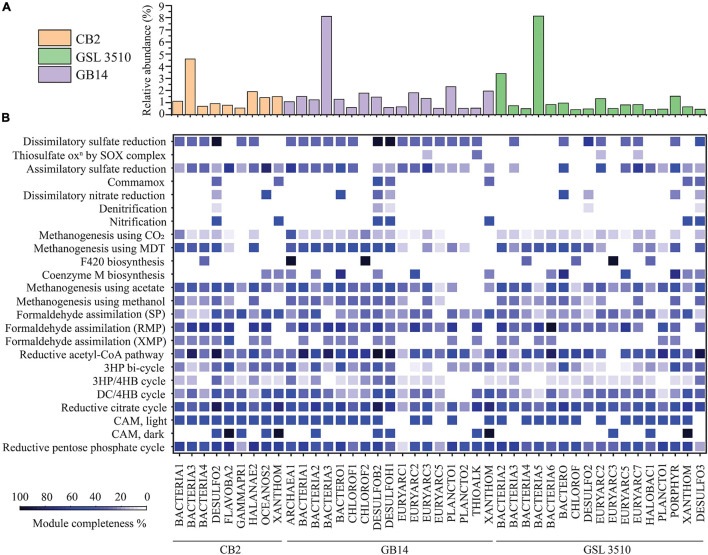
**(A)** The relative abundance of MAGs across the Great Salt Lake sediments is shown as bar graph. **(B)** Heatmap representing the percentage completion of KEGG metabolic modules of MAGs selected for phylogenetic analysis (completeness ≥ 50%, contamination < 5%). (MDT, methylamine–dimethylamine–trimethylamine; SP, serine pathway; RMP, ribulose monophosphate pathway; XMP, xylulose monophosphate pathway; 3HP, 3-hydroxypropionate; 3HP/4HB, 3-hydroxypropionate/4-hydroxybutyrate; DC/4HB, dicarboxylate/4-hydroxybutyrate cycle; CAM, Crassulacean acid metabolism).

A total of 95 MAGS were extracted from the bacterial and archaeal metagenomes of the GSL. Check M v1.0.7 (Parks et al., 2015) was used for the quality assessment of the MAGs. Seventy-six out of the ninety-five MAGs had contamination <5%. The MAGs with contamination ≥5% (19) were manually curated following the MAG refine protocols described in the “Materials and Methods” section. The curation of the contaminated MAGs via splitting resulted in the retention of 33 MAGS with less than 5% contamination. A total of 43 MAGs had completeness greater than 50% and thus were selected for phylogenetic analysis. The genome statistics of the 43 MAGs selected for phylogenetic analysis are shown in Table 2. The genome statistics for the MAGs with contamination <5% and completeness <50% (66) is shown in Supplementary Table S4. The phylogenetic position of the selected MAGs is shown in Figure 4. The relative abundance of the reconstructed MAGs was low (Figure 5A) because only a small proportion of reads (33.9 ± 4.61%) could be mapped back to the MAGs.

**TABLE 2:** Genome statistics of MAGs from the Great Salt Lake (Utah, United States) selected for phylogenetic analysis (completeness ≥ 50%).

MAG ID	Taxonomy	Completeness (%)	Contamination (%)	Bin Size (Mbp)	# Scaffolds
GSL_CB2_BACTERIA1	k__Bacteria	96.7	0	2.48	191
GSL_CB2_HALANAE2	k__Bacteria;p__ Firmicutes;c__ Clostridia_2;o__ Halanaerobiales;f__ Halanaerobiaceae	98.1	0.44	2.16	94
GSL_CB2_GAMMAPR1	k__Bacteria;p__ Proteobacteria;c__ Gammaproteobacteria	67.27	2.65	1.24	244
GSL_CB2_FLAVOBA2	k__ Bacteria;p__ Bacteroidetes;c__ Flavobacteriia;o__ Flavobacteriales;f__ Flavobacteriaceae	96.99	1.52	2.54	190
GSL_CB2_BACTERIA3	k__ Bacteria	64.41	0	1.24	153
GSL_CB2_DESULFO2	k__ Bacteria;p__ Proteobacteria;c__ Deltaproteobacteria;o__ Desulfobacterales;f__ Desulfobacteraceae	96.94	2.74	3.24	265
GSL_CB2_XANTHOM	k__ Bacteria;p__ Proteobacteria;c__ Gammaproteobacteria;o__ Xanthomonadales	95.18	2.52	2.98	152
GSL_CB2_OCEANOS2	k__ Bacteria;p__ Proteobacteria;c__ Gammaproteobacteria;o__ Oceanospirillales	97.27	1.03	3.21	162
GSL_CB2_BACTERIA4	k__ Bacteria	67.8	0	1.5	236
GSL_GSL3510_CHLOROF	k__ Bacteria;p__ Chloroflexi	69.44	2.83	1.87	360
GSL_GSL3510_HALOBAC1	k__ Archaea;p__ Euryarchaeota;c__ Halobacteria;o__ Halobacteriales;f__ Halobacteriaceae	75.15	1.01	1.65	325
GSL_GSL3510_PLANCTO1	k__ Bacteria;p__ Planctomycetes;c__ Planctomycetia;o__ Planctomycetales;f__ Planctomycetaceae	71.49	3.05	3.85	792
GSL_GSL3510_BACTERIA2	k__ Bacteria	77.97	0	1.96	152
GSL_GSL3510_BACTERO	k__ Bacteria;p__ Bacteroidetes;c__ Bacteroidia;o__ Bacteroidales	97.14	4.05	4.72	533
GSL_GSL3510_EURYARC2	k__ Archaea;p__ Euryarchaeota	89.6	1.6	1.71	114
GSL_GSL3510_BACTERIA3	k__ Bacteria	97.8	0	2.48	229
GSL_GSL3510_BACTERIA4	k__ Bacteria	64.87	3.95	1.61	318
GSL_GSL3510_EURYARC3	k__ Archaea;p__ Euryarchaeota	72.98	4.8	0.82	123
GSL_GSL3510_BACTERIA5	k__ Bacteria	55.93	0	0.76	100
GSL_GSL3510_BACTERIA6	k__ Bacteria	79.66	0	2.92	316
GSL_GSL3510_EURYARC5	k__ Archaea;p__ Euryarchaeota	91.2	2.4	1.75	189
GSL_GSL3510_XANTHOM	k__ Bacteria;p__ Proteobacteria;c__ Gammaproteobacteria;o__ Xanthomonadales	93.1	4.57	2.88	323
GSL_GSL3510_EURYARC7	k__ Archaea;p__ Euryarchaeota	94.4	0.8	2.11	112
GSL_GSL3510_DESULFO2	k__ Bacteria;p__ Proteobacteria;c__ Deltaproteobacteria;o__ Desulfobacterales;f__ Desulfobacteraceae	56.86	0.06	1.63	287
GSL_GSL3510_PORPHYR	k__ Bacteria;p__ Bacteroidetes;c__ Bacteroidia;o__ Bacteroidales;f__ Porphyromonadaceae	94.89	2.96	3.09	158
GSL_GSL3510_DESULFO3	k__ Bacteria;p__ Proteobacteria;c__ Deltaproteobacteria;o__ Desulfobacterales;f__ Desulfobacteraceae	66.97	3.23	2.05	388
GSL_GB14_PLANCTO1	k__ Bacteria;p__ Planctomycetes;c__ Planctomycetia;o__ Planctomycetales;f__ Planctomycetaceae	96.59	0	2.74	82
GSL_GB14_EURYARC1	k__ Archaea;p__ Euryarchaeota	55.83	1.6	0.84	189
GSL_GB14_DESULFOH1	k__ Bacteria;p__ Proteobacteria;c__ Deltaproteobacteria;o__ Desulfovibrionales;f__ Desulfohalobiaceae	71.65	1.03	2.19	504
GSL_GB14_EURYARC2	k__ Archaea;p__ Euryarchaeota	90	1.6	1.78	121
GSL_GB14_BACTERIA1	k__ Bacteria	84.75	1.69	2.39	201
GSL_GB14_ARCHAEA1	k__ Archaea (root)	93.93	4.67	2.94	176
GSL_GB14_CHLOROF1	k__ Bacteria;p__ Chloroflexi	63.64	4.99	2.41	525
GSL_GB14_EURYARC3	k__ Archaea;p__ Euryarchaeota	91.44	3.6	1.75	147
GSL_GB14_XANTHOM	k__ Bacteria;p__ Proteobacteria;c__ Gammaproteobacteria;o__ Xanthomonadales	94.89	2.64	2.98	129
GSL_GB14_CHLOROF2	k__ Bacteria;p__ Chloroflexi	84.15	4.55	2.47	204
GSL_GB14_BACTERIA2	k__ Bacteria	91.91	4.27	2.54	107
GSL_GB14_PLANCTO2	k__ Bacteria;p__ Planctomycetes;c__ Planctomycetia;o__ Planctomycetales;f__ Planctomycetaceae	52.92	3.62	2.26	508
GSL_GB14_EURYARC5	k__ Archaea;p__ Euryarchaeota	56.36	1.6	1.06	261
GSL_GB14_BACTERO1	k__ Bacteria;p__ Bacteroidetes;c__ Bacteroidia;o__ Bacteroidales	95.71	4.37	4.64	429
GSL_GB14_THIOALK	k__ Bacteria;p__ Proteobacteria;c__ Gammaproteobacteria;o__ Chromatiales;f__ Ectothiorhodospiraceae;g__ Thioalkalivibrio;s__ Thioalkalivibrio_thiocyanoxidans	60.69	4.2	1.71	404
GSL_GB14_BACTERIA3	k__ Bacteria	63.56	0	1.18	164
GSL_GB14_DESULFOB2	k__ Bacteria;p__ Proteobacteria;c__ Deltaproteobacteria;o__ Desulfobacterales;f__ Desulfobacteraceae	98.06	1.61	3.29	99

Prokka v 1.14.6 (Seemann, 2014) was used for gene/ORF prediction and annotation of the 109 refined MAGs. The metabolism of the MAGs was assessed based on the completeness of different metabolic pathways (KEGG Pathway). The nitrogen, sulfur, methane, and carbon fixation pathways were studied in detail for 43 MAGs selected for phylogenetic analysis to elucidate the nutrient cycling in the GSL (Figure 5B). The findings are presented in the following sections.

#### Carbon Fixation

Ten GSL MAGs showed a substantially complete (>70% complete) reductive acetyl-CoA module (Wood–Ljungdahl pathway) across the GSL (Figure 5B). At all three sites, MAGs of *Desulfobacteraceae* (CB2_DESULFO2, GSL3510_DESULFO3, GB14_DESULFOB2) were observed to take part in the reductive acetyl-CoA pathway. Similarly, a MAG classified as member of *Desulfohalobiaceae* from site GB14 (GB14_DESULFOH1) and another MAG classified as member of *Planctomycetaceae* in site GSL 3510 (GSL3510_PLANCTO1) contained more than 70% complete reductive acetyl-CoA pathway (Figure 5B). Only two MAGs assigned to *Flavobacteriaceae* (CB2_FLAVOBA2) and *Halanaerobiaceae* (CB2_HALANAE2) from site CB2 showed a substantially complete module (≥70%) for the reductive pentose phosphate cycle (Calvin–Benson–Bassham cycle). Although the reductive pentose phosphate cycle is the predominating CO_2_ fixation pathway globally (Wolfe and McBride, 1971; Pride et al., 2003; Bergmann et al., 2005), the anoxia within the sediments of the GSL supports the reductive acetyl-CoA pathway. These results suggest that the reductive acetyl-CoA pathway may be the primary pathway for carbon fixation in the GSL sediments.

#### One Carbon Metabolism

Metagenome-assembled genomes of *Chloroflexi* (GB14_CHLOROF1, GB14_CHLOROF2) contained at least half of the required methanogenesis genes using acetate and methylamine–dimethylamine–trimethylamine substrate, respectively (Figure 5B). MAGs of *Desulfobacteraceae* (CB2_DESULFO2, GB14_DESULFOB2), *Desulfohalobiaceae* (GB14_DESULFOH1), *Bacteroidales* (GB14_BACTERO1, GSL3510_BACTERO), *Halanaerobiaceae* (CB2_HALANAE2) contained genes for methanogenesis using both acetate and methylamine–dimethylamine–trimethylamine (Figure 5B). MAGs of *Chloroflexi* (GB14_CHLOROF2) and *Euryarchaeota* (GB14_ARCHAEA1) contained enzymes for F420 synthesis, an essential catabolic cofactor in methanogens. Similarly, MAGs of *Bacteroidales* (GB14_BACTERO1, GSL3510_BACTERO), *Euryarchaeota* (GB14_EURYARC2, GSL3510_EURYARC5), and *Porphyromonadaceae* (GSL3510_PORPHYR) contained genes coding for coenzyme M biosynthesis. Coenzyme M plays a vital role in methanogenesis by functioning as a C1 carrier Wolfe and McBride, 1971).

Metagenome-assembled genomes of *Desulfobacteraceae* (CB2_DESULFO2, GB14_DESULFOB2), *Flavobacteriaceae* (CB2_FLAVOBA2), *Halanaerobiaceae* (CB2_HALANAE2), *Oceanospirillales* (CB2_OCEANOS2), *Bacteroidales* (GB14_BACTERO1), *Euryarchaeota* (GB14_EURYARC2, GSL3510_EURYARC5), *Planctomycetaceae* (GB14_PLANCTO1) contained more than two-thirds of genes responsible for formaldehyde assimilation via the ribulose monophosphate pathway. None of the MAGs showed a substantial complete module (>50%) for formaldehyde assimilation via serine and xylulose monophosphate pathways (Figure 5B).

#### Sulfur Metabolism

The GSL sediments harbor a diverse population of sulfate reducers. *Desulfobacteraceae* at sites CB2 and GB14 and *Desulfohalobiaceae* at site GB14 are dissimilatory sulfate reducers of GSL. Some MAGs of *Desulfobacteraceae* (CB2_DESULFO2, GB14_DESULFOB2) and *Desulfohalobiaceae* (GB14_DESULFOH1) contained all the necessary genes for a dissimilatory sulfate reduction lifestyle (Figure 5B). In addition, *Desulfobacteraceae* (GSL3510_DESULFO2), sulfate reducer from the site GSL 3510, have genes of dissimilatory sulfate reduction pathway (66.67% of KEGG module completion). The MAGs of *Flavobacteria* (CB2_FLAVOB2) and *Oceanospirillales* (CB2_OCEANOS2) from the site CB2 and *Euryarchaeota* from the sites GSL 3510 (GSL3510_EURYARC2, GSL3510_EURYARC7) and GB14 (GB14_EURYARC3) have at least half of the genes for assimilatory sulfate reduction. MAGs of *Bacteroidales* (GSL3510_BACTERO, GB14_BACTERO1) and *Porphyromonadaceae* (GSL3510_PORPHYR) also contained substantially complete modules for assimilatory sulfate reduction. MAGs of *Euryarchaeota* and *Thioalkalivibrio* contained few genes for thiosulfate oxidation (<50% complete KEGG modules) (Figure 5B).

#### Nitrogen Metabolism

Bacteria and archaea are involved in nitrogen cycling connect carbon, phosphorous, and sulfur metabolism. Nitrogen metabolizing bacteria and archaea have been shown to be present in the GSL ecosystem (Motlagh et al., 2017). Multiple phylogenetically classified refined MAGs from the GSL possessed genes for nitrification (7), denitrification (5), and dissimilatory nitrate reduction (9) (Figure 5B).

The *Xanthomonadales* MAGs were found across GSL sediments. MAGs of *Xanthomonadales* (CB2_XANTHOM, GSL3510_XANTHOM, GB14_XANTHOM) contained *hao* gene but lack genes that encode ammonia mono-oxygenase subunits for nitrification. Sulfate reducers *Desulfobacteraceae* (CB2_DESULFO2, GB14_DESULFOB2, GSL3510_DESULFO3) and *Desulfohalobiaceae* (GB14_DESULFOH1) have a *hao* gene that encodes a potentially functional HAO protein. The HAO protein is not involved in nitrification but a result of horizontal gene transfer (Bergmann et al., 2005; Figure 5B). A MAG of *Desulfobacteraceae* (GB14_DESULFOB2) additionally showed a nitrite oxidoreductase gene for converting nitrite to nitrate. MAGs of *Bacteroidales* and *Oceanospirillales* had half of the genes required for dissimilatory nitrate reduction. MAGs of *Bacteroidales* (GB14_BACTERO1; GSL3510_BACTERO) contained *nrf*A and *nrf*H genes, while MAG of *Oceanospirillales* (CB2_OCEANOS2) was detected with *nir*B and *nir*D genes.

The absence of genes can also be due to the incomplete construction of MAGs.

### Viral Ecology of the Great Salt Lake Sediments

The metavirome analysis showed that the viruses belonging to the *Siphoviridae* family of order *Caudovirales* were dominant across the GSL (39.12 ± 19.8%) (Figure 2B). Nearly 60.42% of the viruses at the anoxic site CB2 in the GSL belonged to the *Siphoviridae* family. Viruses assigned to *Myoviridae* (13.7 ± 6.6%) and *Podoviridae* (5.43 ± 0.64%) were the other dominant families within GSL viromes. *Siphoviridae*, *Myoviridae*, and *Podoviridae* are dominant viral families in the aquatic ecosystems (Gong et al., 2018; Kallies et al., 2019). One-third (33.15 ± 20.3%) of the total viruses across the GSL were categorized as unclassified DNA viruses (Figure 2B). At the site GSL 3510, over half (54.77%) of the total viruses were unclassified DNA viruses. This is due to the under representation of taxonomically classified viruses from hypersaline ecosystem in reference databases such as GenBank or RefSeq. *Haloviruses*, viruses that infect halophilic archaea, were found in the GSL sediments. The *Haloviruses* at sites CB2, GSL 3510, and GB14 comprised 14.23%, 52.6%, 29.39%, respectively, of total viral abundance. Viruses of genus *Peduovirus* were detected at two sites, GSL 3510 (5.54%), GB14 (11.2%). Additionally, *Nonlabens* virus P12024L were also recognized at three sites, CB2 (6.65%), GB14 (1.76%) and GSL 3510 (0.3%).

Comparison of IMG/VR database (Paez-Espino et al., 2019) showed that 67.41 and 42.12% of viruses from the sites GSL 3510 and GB14 had been previously identified in hypersaline environments (Figure 2C). Saline viruses were also observed in sediments from GSL 3510 and GB14, 5.38 and 12.02%, respectively. At site CB2, the abundance of viruses from hypersaline and saline, 28.57 and 25.44%, respectively, were similar (Figure 2C).

Post metavirome assembly with SPAdes v3.13.0 (Bankevich et al., 2012) and Prodigal v2.6.3 (Hyatt et al., 2010) for gene/ORF prediction and annotations, we used SUPER-FOCUS (Silva et al., 2016) for functional prediction. As anticipated, for viral genomes, a significant proportion of the predicted viral ORF annotations were phages, prophages, transposable elements, plasmids (Figure 2D). Genes of the stress response were also identified within the viral ORFs. The stress response genes included genes encoding cold shock proteins and sigmaB stress response regulation. The ORFs identified from the CVCs (Supplementary Figure S2) were also annotated with Prokka v1.14.6 (Seemann, 2014). Genome map of the viral genomes with auxiliary metabolic genes (AMGs) are shown in Supplementary Figure S3. AMGs *psbA*; *rbcL*, *cbbL*; gene encoding SHMT and nitric oxide reductase *NorQ* were identified across the GSL. Viruses in CB2 contained *psbA* gene for the Photosystem II module and *rbcL, cbbL* gene involved in reductive pentose phosphate cycle (Calvin cycle) for carbon fixation. Gene encoding SHMT was observed in viral genomes from CB2 and GSL 3510 sediments. SHMT is an enzyme involved in the assimilation of formaldehyde into intermediates of central metabolic pathways. Gene encoding nitric oxide reductase *NorQ* protein was observed in viral genomes from GSL 3510 sediments. *Nor*Q is a membrane protein required in denitrification (Kahle et al., 2018).

Based on the lifestyle assessment of the viruses using BACPHLIP (Hockenberry and Wilke, 2021), a total of 68 viruses were identified as lysogenic out of 3316 viral genomes (Supplementary Table S9). These predictions should be interpreted with caution because many sequences are incomplete genomes which can underestimate which viruses are lysogenic.

### Identification of Lysogenized Metagenome-Assembled Genomes in the Great Salt Lake Sediments

Contigs within the refined MAGs were checked for viral signal through Virsorter (Roux et al., 2015). A total of 43 and 103 contigs of viral origin were identified within 28 and 11 MAGs, respectively, extracted from the RMC and RVC (Supplementary Figure S2). The MAGs with viral contigs were referred as lysogens. The lysogens in the GSL accounted for 17.52 (±3.75)% of the bacterial and archaeal community (Supplementary Figure S5). The taxonomic lineage of the lysogenic MAGs was obtained with CheckMv1.0.7 (Parks et al., 2015). The taxonomic affiliation of contigs of viral origin was determined by results of tBLASTx using reference sequence database for viruses (RefSeq-viral database), National Center for Biotechnological Information (NCBI) (accessed on September 2020). Supplementary Table S5 shows the putative lysogenic virus–host associations identified.

*Flavobacterium* virus FCL2, *Nonlabens* virus P12024L, and *Pseudomonas* virus KPP25 were associated with bacterial MAGs of family *Flavobacteriaceae* from site CB2. Similarly, viruses of family *Siphoviridae* and *Podoviridae* were associated with bacterial MAGs of the order *Bacteroidales* from site GSL 3510 and GB14 (Supplementary Table S5). MAG of family *Planctomycetaceae* was observed to form a lysogenic association with the *Myoviridae* family virus (Supplementary Table S5). Across the GSL, 14.45%, 16.41%, 21.7% of the refined MAGs, respectively, were lysogenic in sites CB2, GSL 3510, and GB14 (Supplementary Figure S5). Additionally, the viruses’ bacterial and archaeal hosts were also identified with the Virus–Host database (Mihara et al., 2016).

The RVCs were binned into genomes with metaBAT v2.12.1 (Kang et al., 2019). The MAGs were checked if they contained contigs of viral origin. A total of 11 MAGs (Supplementary Table S6) contained viral genomes within it, and thus they were also termed lysogenic MAGs. Viruses of genus *Hapunavirus*, *Peduovirus*, *Lessievirus*, *Kleczkowskavirus*, and *Detrevirus* formed a lysogenic association with MAGs of genus *Pseudomonas*. Viruses belonging to *Podoviridae*, *Escherichia virus mEpX2*, and *Siphoviridae* were found in the MAG genus *Stenotrophomonas.* Virus belonging to genus *Salterprovirus* was associated with the MAG of genus *Microbacterium*.

### CRISPRs of Great Salt Lake Bacterial and Archaeal Contigs

A total of 8, 16, and 21 valid CRISPR arrays with 29, 79, and 109 spacer sequences were predicted in bacterial and archaeal contigs of the cellular fraction from CB2, GSL 3510, and GB14 (Supplementary Figure S6). All the spacer sequences were unique at 97% nucleotide level similarity.

The CRISPR spacers identified in bacterial and archaeal sequences were linked to the viral genomes with BLASTn. Thirty-one putative virus–host associations were established (minimum percentage identity: 90% with length ≥ 15 bp, maximum mismatch of one nucleotide, and e-value of 0.1) (Supplementary Table S7). The CRISPR spacer identified in a contig of *Marinilabilia* from phylum *Bacteroidetes* matched the virus belonging to the family *Siphoviridae* with 100% query coverage. This was the only association with 100% query coverage. The average query coverage was low (49.29 ± 12.45%, mean ± SD). The spacer sequences were also matched with the reference viral genome (RVG) and environmental viral genome (EVG) databases (see the section “Taxonomic Classification and Open Reading Frame Prediction of Viral, Bacterial, and Archaeal Sequences”). A total of twenty-three virus–host associations were determined against the RVG database but no associations exhibiting query coverage greater than 95% were observed (average query coverage: 58.43 ± 9.31%). CRISPR spacers in the bacterial and archaeal sequences matched with viruses from marine ecosystems (Supplementary Figure S7).

### Bacterial and Archaeal Host of the Viruses From the Great Salt Lake

Bacterial and Archaeal hosts for 151 putative GSL viruses were assigned to bacterial and archaeal genomes in the NCBI RefSeq database based on (a) homology matches, (b) shared tRNAs, and (c) CRISPR spacers (Supplementary Table S8). The majority of predicted hosts were Bacteria (142) with few Archaea (9). Among the 151 predicted virus–host pairings, *Proteobacteria* (54.3%), *Firmicutes* (16.56%), and *Actinobacteria* (15.89%) were the dominant hosts of the GSL viruses. Other hosts of the putative viruses were *Euryarchaeota* (8), *Bacteroidetes* (3), *Acidobacteria* (2), *Fusobacteria* (2), *Tenericutes* (2), *Crenarchaeota* (1), *Chlorobi* (1), *Chloroflexi* (1).

## Discussion

### Microbial and Viral Community Composition in the Great Salt Lake Is Driven by Nutrients

Spherical, head-tail, and lemon-shaped viral morphotypes have been previously identified in hypersaline environments such as solar salterns and salt flats (Garcia-Heredia et al., 2012; Santos et al., 2012; Ramos-Barbero et al., 2019). Lemon-shaped viruses are generally known to infect archaea, while spherical and head-tail viruses can infect bacteria and archaea (Atanasova et al., 2015). Viruses of family *Siphoviridae*, *Myoviridae*, and *Podoviridae* order *Caudovirales* are dominant among GSL sediments (Figure 2B). The GSL viral communities are similar to the viruses of hypersaline ponds/Lakes with varying salinity (8 to 36%) (Roux et al., 2016; Motlagh et al., 2017). A notable proportion of viral genomes (33.15 ± 20.3%) were identified as unclassified DNA viruses, presumably because the available database lacks a comprehensive catalog of viruses from hypersaline environments. The limitation is thought to conceal the viral diversity in the less studied extreme ecosystems such as the GSL (Atanasova et al., 2015; Dávila-Ramos et al., 2019; Ramos-Barbero et al., 2019). The current study elucidates the virosphere from underrepresented hypersaline GSL, an extreme environment. This study’s viral diversity will add to the growing public repositories of viral genome databases and facilitate hypersaline virus research.

Great Salt Lake sites GSL 3510 and CB2, with a salinity of 12.14% and 17.59% (Table 1), respectively, harbors viruses of saline and hypersaline environments. More than two-thirds (72.79%) of the total viral abundance at site GSL 3510 are known saline and hypersaline viruses (Figure 2C). At the site CB2, a little over half (54.01%) of the total viruses are known to be from saline and hypersaline environments (Figure 2C). Although the salinity increases between the GSL sites, GSL 3510 and CB2, the abundance of previously known viruses from saline and hypersaline environments decreases. The GSL site CB2 harbors viruses that previously were not known to be in hypersaline environments.

Viruses from saline and hypersaline environments are known as *Haloviruses* (Emerson et al., 2012; Santos et al., 2012). These constituted one-third (32.07 ± 19.33%) of the total viral community across the GSL. *Haloviruses* infect halophilic archaea (Atanasova et al., 2015). However, the abundance of *Haloviruses* of GSL does not change with increased salinity across the Lake (Figure 2B). The GSL site GSL 3510, with a salinity of 12.14%, harbors *Haloviruses*. More than half (52.6% abundance) of the total virosphere at site GSL 3510 are *Haloviruses*. Whereas the GSL site CB2, with a high salinity of 17.59%, has *Haloviruses* but less abundant (14.23%). The *Haloviruses* abundance across the GSL is in congruence with its host, halophilic archaea. At the GSL sites GSL 3510 and CB2, the halophilic archaeal abundance was 89.74 and 19.6%, respectively, of the total bacterial and archaeal abundance (Supplementary Figure S4A).

In the GSL ecosystem, the abundance of *Haloviruses* and their host, halophilic archaea, are not in congruence with the salinity gradient. This contrasts with Roux et al. (2016) findings that across hypersaline environments, the abundance of *Haloviruses* and their archaeal hosts increase with salinity (Roux et al., 2016). Salinity alone does not affect the viral, bacterial, and archaeal diversity across the hypersaline GSL. In addition to salinity, inorganic nutrients and available organic carbon affect bacterial and archaeal diversity. Among three GSL sites, site CB2 is rich in nutrients (Table 1) and has high salinity. This site harbors a diverse [Shannon diversity index (H), 0.89] bacterial and archaeal community. The availability of nutrients as organic carbon and inorganics supports a diverse viral, bacterial, and archaeal community at the GSL site CB2. Our results suggest that the viral, bacterial, and archaeal community composition in the southern arm of the GSL along the salinity gradient is primarily influenced by concentrations of organic carbon and inorganic nutrients, while salinity is a secondary factor.

### Reductive Acetyl-CoA Pathway Is the Primary Carbon Fixation Pathway in the Great Salt Lake Sediments

Our study found ten MAGs including those classified as *Desulfobacteraceae*, *Desulfohalobiaceae*, *Planctomycetaceae*, encoding genes involved in the reductive acetyl-CoA, or WL pathway in GSL sediments (Figure 5B). In all three sites, most of the genomes with substantially complete reductive acetyl-CoA pathway had genes encoding for CO dehydrogenase/acetyl-CoA synthase (Figure 5B). CO dehydrogenase/acetyl-CoA synthase is a critical enzyme in the WL pathway to reduce CO_2_ to CO and synthesize acetyl-CoA from the methyl carbonyl residues (Pezacka and Wood, 1984; Ragsdale and Wood, 1985). This indicates that the reductive acetyl-CoA pathway (WL pathway) is a primary carbon fixation channel in the GSL sediments. Recognized sulfate reducers *Desulfobacteraceae* and *Desulfohalobiaceae* in the GSL ecosystem (Brandt and Ingvorsen, 1997; Jakobsen et al., 2006; Kuever, 2014a, b) were identified with genes required for dissimilatory sulfate reduction as well as the WL pathway (Figure 5B). This suggests that *Desulfobacteraceae* and *Desulfohalobiaceae* function via coupled exergonic sulfate reduction and endergonic acetate oxidation (reverse WL pathway), as reported before (Schauder et al., 1988; Spormann and Thauer, 1988; Ferry, 1992). In our study, the MAGs of sulfur oxidizers *Oceanospirillales* and *Thioalkalivibrio thiocyanoxidans* showed minimal or no genes responsible for the thiosulfate oxidation pathway (Figure 5B), although previous studies have identified the presence of that pathway in these genomes (Swan et al., 2011; Berben et al., 2017). The genomes of strict anaerobes *Desulfobacteraceae* and *Desulfohalobiaceae* displayed partially complete nitrification pathways. However, the possibility of nitrification within these anaerobes cannot be asserted due to the absence of *ammonia mono-oxygenase subunits* in the MAGs. This suggests that the nutrient cycling pathway in GSL sediments observed in our genome-based study is still not comprehensive.

### Lysogenic Metagenome-Assembled Genomes and Expanding Viral Host Range

In the GSL ecosystem, sequence reads that belong to phylum *Euryarchaeota*, and *Proteobacteria* has higher abundance of the total bacteria and archaea, respectively (Figure 3A). Across the GSL, we predicted eight viruses that infect MAGs affiliated to phylum *Euryarchaeota* (Supplementary Table S8). Such low numbers for viruses of *Euryarchaeota* are due to limitations in current viral-host prediction computational analysis. More than half (54.3%) of the 151 putative GSL viruses-host pairing were to MAGs of the phylum *Proteobacteria* (Supplementary Table S8).

The genetic content between the GSL viruses and the bacterial and archaeal genomes from the NCBI RefSeq database was compared based on homology matches, shared tRNAs, and CRISPR spacers. The prediction of the bacterial and archaeal hosts of the GSL viruses shows the association between four bacterial and archaeal genomes (lysogenic MAGs) with five GSL viruses are virus–host associations. These GSL viruses infect the bacteria and archaea that they co-binned with [Genus *Stenotrophomonas* (2), genus *Pseudomonas* (1), family *Rhodobacteraceae* (1), family *Halanaerobiaceae* (1)]. However, the host of two viruses co-binned with the MAGs of phylum *Bacteroidetes* and phylum *Proteobacteria* were genomes of phylum *Firmicutes*. The differences between the association between virus, bacteria, and archaea in our study and the virus–host prediction indicate expanding host range of the GSL viruses.

### Great Salt Lake Viruses Encode Auxiliary Metabolic Genes Involved in Biogeochemical Cycles

The hypersaline viruses of GSL have AMGs. These AMGs could have been acquired from their immediate host during infection events (Mann et al., 2003; Thompson et al., 2011; Crummett et al., 2016). The historical acquisition of genes has been studied in cyanophage genomes. It has been shown that the selective pressure leads to incorporation of host-like genes in the viral genomes as Calvin cycle inhibitor CP12 was identified in cyanophage genomes to redirect host metabolism toward DNA biosynthesis. Also, there was a homology observed between the DNA sequences from bacteriophage S-PM2 and host *Synechococcus* encoding D1 proteins suggesting the horizontal acquisition of genes into the viral genome from their host (Thompson et al., 2011). These gene/s modulate host metabolism during infection events for the viruses to replicate efficiently (Breitbart et al., 2007). Viruses identified as *Enterococcus phage EF24C*, *Lactococcus phage phiL47* (CB2) and *Aeromonas virus 31* (GSL3510) encoding SHMT were identified. The SHMT enzyme is required for formaldehyde assimilation via the serine pathway (Chistoserdova et al., 1994). This enzyme catalyzes the reaction of formaldehyde and glycine for the formation of L- serine. Bacterial and archaeal host for the GSL virus with SHMT gene is unknown. The viruses with AMG SHMT likely acquired it from bacterial/archaeal host during previous infection event.

A viral genome co-binned with *Halodesulfurarchaeum* MAG (lysogenic archaea) displayed an AMG which encodes nitric oxide reductase (*Nor*Q) protein. Nitric oxide reductase (*Nor*Q) facilitates the insertion of non-heme Fe (Fe_*B*_) into the cytochrome c dependent nitric oxide reductase (cNOR)- a vital membrane protein required for denitrification (Kahle et al., 2018). However, the virus may have acquired the AMG during past infection of the GSL denitrifier/s. *Halodesulfurarchaeum* depends on sulfur for respiration (Sorokin et al., 2017). In addition, the GSL draft *Halodesulfurarchaeum* MAG (GSL_GSL3510_HALOBAC1; 75.15% complete) lacks the genes for denitrification.

Viruses in GSL were also identified with AMGs responsible for carbon fixation and photosynthesis. The *psbA* gene required for Photosystem II and *rbcL, cbbL* gene involved in reductive pentose phosphate cycle (Calvin cycle) were detected within viruses from GSL site CB2. The *Cyanobacteria* MAG with photosynthetic metabolism and substantially complete Calvin cycle pathway from site CB2 had no association with the virus. We suspect that most sequences (66.1 ± 4.61%) did not get binned as draft bacterial and archaeal genomes. This led to failure to properly reconstruct the virus–host infection network of GSL. Without knowing the bacterial and archaeal host to these viruses, it is difficult to predict how these metabolic genes contribute to different biogeochemical processes. Nevertheless, the AMGs have ecological significance as they can be expressed or recombined with the host genes during infection thus altering host metabolisms.

## Conclusion

Microbes (algae, bacteria, and archaea) are the primary producers in the GSL. They support other aquatic life forms such as Brine shrimps. Bacteria and archaea perform critical ecosystem functions by cycling nutrients (N, P, S, and C) (Motlagh et al., 2017). These microbes provide biological foundations upon which other unicellular and multi-cellular organisms establish themselves. Bacterial and archaea that inhabit the GSL extreme environments are often endemic to the region. The viruses of GSL that infect bacteria and archaea decide the fate of the host cell. During infection events, the viruses reprogram the host cellular metabolism for replication and propagation (Forterre, 2013; Rosenwasser et al., 2016). One-third of the GSL viruses are unclassified DNA viruses comprising *Haloviruses*. Two-third of the viruses belong to the order *Caudovirales* and spread across the families *Siphoviridae*, *Myoviridae*, and *Podoviridae*. The *Haloviruses* that infect halophilic archaea are low in numbers at the site with higher salinity, inorganic nutrients, and organic carbon. Diverging of the Roux et al. (2016) findings, the abundance of *Haloviruses* and its host are independent of salinity changes across the lake. In GSL, salinity alone does not determine the viral, bacterial, archaeal diversity. Instead, organic carbon, inorganic nutrients, and salinity fuel the bacterial and archaeal diversity across the lake. In addition, the microbes are equipped with genes for Calvin–Benson–Bassham (aerobic), and WL (anaerobic), carbon fixation pathways. The metabolic flexibility by two carbon fixation pathways supports bacterial and archaeal growth. The viruses that infect bacteria and archaea replicate and propagate by lysing the host. Lysogenic infection events are rare at this site (Supplementary Figure S5). The nutrient abundant regions of GSL support lytic lifestyle for the viruses. In GSL, the acetyl-coA pathway is the primary carbon fixation pathway. Sulfate reducers *Desulfobacteraceae* and *Desulfohalobiaceae* are prevalent across the GSL.

This study’s findings will provide important information regarding growth conditions and nutritional requirements for the isolation of a diverse set of bacteria, archaea, and viruses. This information will facilitate the culture-based study of GSL microbiome and viruses, thus elucidating their roles in biogeochemical cycling through detailed physiological studies.

## Data Availability Statement

All DNA sequence FASTQ files are deposited in the NCBI Nucleotide Archive under SRR14023866 (CB2 Bacterial and Archaeal), SRR 14023937 (GSL 3510 Bacterial and Archaeal), SRR14023877 (GB14 Bacterial and Archaeal), SRR14023878 (CB2 Viral), SRR14023865 (GSL3510 Viral), and SRR14023938 (GB14 Viral). The Bioproject id is PRJNA714934. The NCBI genome accession numbers for the MAGs and viral assemblies are shown in Supplementary Table S10.

## Author Contributions

BB conducted experiments, bioinformatic analysis, sequence data management, wrote the manuscript, and submissions to public repository. AB conducted bioinformatic analysis and assisted in writing the manuscript. FC contributed toward bioinformatic analysis (viral–host analysis, gene prediction and phage identification). RG supervised the GSL research, mentored students, and finalized the manuscript for submission.

## Conflict of Interest

The authors declare that the research was conducted in the absence of any commercial or financial relationships that could be construed as a potential conflict of interest.

## Publisher’s Note

All claims expressed in this article are solely those of the authors and do not necessarily represent those of their affiliated organizations, or those of the publisher, the editors and the reviewers. Any product that may be evaluated in this article, or claim that may be made by its manufacturer, is not guaranteed or endorsed by the publisher.

## Supplementary Material

Supplementary Figure S1 Vertical profile of physio-chemical data (specific conductivity, dissolved oxygen and oxidation–reduction potential) in three Great Salt Lake (GSL) study areas [**(A)** CB2, **(B)** GSL 3510, **(C)** GB14].

Supplementary Figure S2 Workflow schematic of the viral, bacterial, and archaeal metagenome analysis.

Supplementary Figure S3 Genome map of viral genomes (contigs) identified from the Great Salt Lake sediments with auxiliary metabolic genes (AMGs). The AMGs are denoted by asterisk (*). Arrow length in the genome maps is proportional to the size of the open reading frame (ORF).

Supplementary Figure S4 Taxonomic profiling of the bacterial and archaeal communities in sediments across the Great Salt Lake in **(A)** Kingdom, **(B)** Class, **(C)** Order, **(D)** Family, **(E)** Genus, and **(F)** Species level.

Supplementary Figure S5 Relative abundance of lysogenic MAGs represented by bar graphs.

Supplementary Figure S6 Number of CRISPR arrays and spacer sequences in bacterial and archaeal contigs (per million reads) across the Great Salt Lake.

Supplementary Figure S7 Association of CRISPR spacers from the bacterial and archaeal contigs with the viruses from the marine, saline, and hypersaline aquatic ecosystems (extracted from IMG/VR database).

Supplementary Figure S8 Comparison of relative abundance between bacteria and archaeal phyla (based on bacterial and archaeal metagenome reads) with viruses that infects them (based on the Virus–Host database) across the Great Salt Lake sediments.

Supplementary Table S1 Metagenomic read statistics.

Supplementary Table S2 *De novo* assembly statistics of virome, bacterial, and archaeal metagenome.

Supplementary Table S3 Summary of the number of viral contigs identified using VirSorter.

Supplementary Table S4 Genome statistics of MAGs from the Great Salt Lake (Utah, United States) (completeness < 50%).

Supplementary Table S5 Lysogenic MAGs identified in the bacterial and archaeal sequences.

Supplementary Table S6 Lysogenic MAGs identified in the viral sequences.

Supplementary Table S7 Summary of virus–host association based on CRISPR array.

Supplementary Table S8 Computational host prediction of the viral contigs identified against the bacterial and archaeal genomes in the NCBI RefSeq database based on homology matches, shared tRNAs, and CRISPR spacers.

Supplementary Table S9 Lifestyle prediction of viruses based on the conserved protein domains using BACPHLIP (Hockenberry and Wilke, 2021).

Supplementary Table S10 NCBI accession numbers of the Great Salt Lake metagenome assembled genomes (MAGs) and viral assemblies.

## Abbreviations

GSL: Great Salt Lake
MAG: metagenome assembled genome
AMG: auxiliary metabolic gene
USGS: United States Geological Survey
ORP: oxidation–reduction potential
DO: dissolved oxygen
MWCO: molecular weight cut-off
RASL: random amplified shotgun library
LASL: linker-amplified shotgun library
MDA: multiple displacement amplification
RMC: raw metagenome contigs
RVC: raw metavirome contigs
CMC: curated metagenome contigs
CVC: combined viral contigs
RVG: reference viral genomes
EVG: environmental viral genomes
ORF: open reading frame
NCBI: National Center for Biotechnology Information
LCB: locally collinear blocks
SHMT: glycine hydroxymethyltransferase
GTDB: Genomic Taxonomy Database
TOC: total organic carbon
TEM: transmission electron microscopy
WL: Wood–Ljungdahl.
