# Supplementary figures and images for: Viruses and Their Interactions With Bacteria and Archaea of Hypersaline Great Salt Lake
Source: Front Microbiol. 2021 Sep 28;12:701414. doi: 10.3389/fmicb.2021.701414 (PMC8506154; doi:10.3389/fmicb.2021.701414)

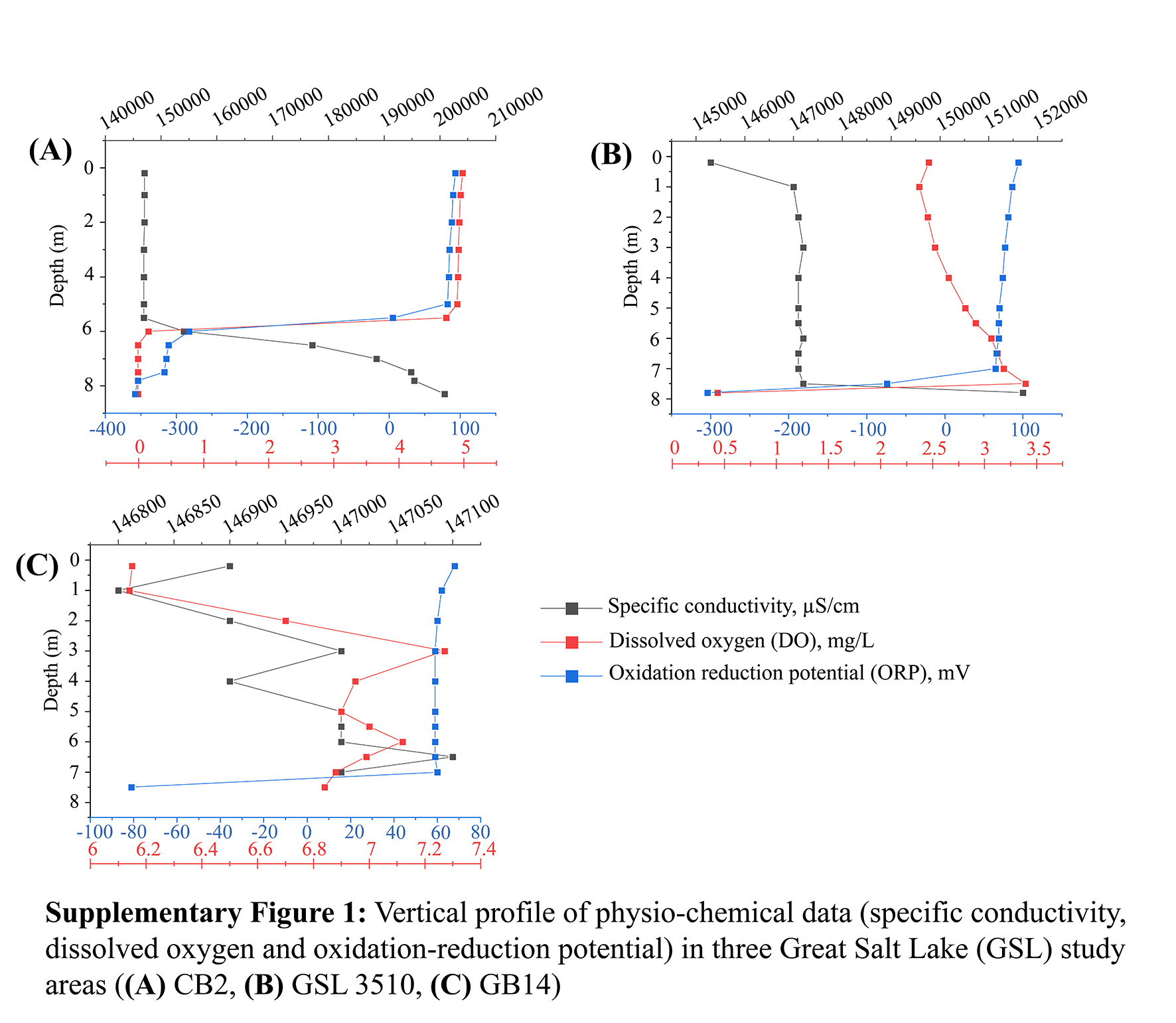

Supplement: Supplementary Figure S1 — Vertical profile of physio-chemical data (specific conductivity, dissolved oxygen and oxidation–reduction potential) in three Great Salt Lake (GSL) study areas [(A) CB2, (B) GSL 3510, (C) GB14]. [file Image_1.JPEG]

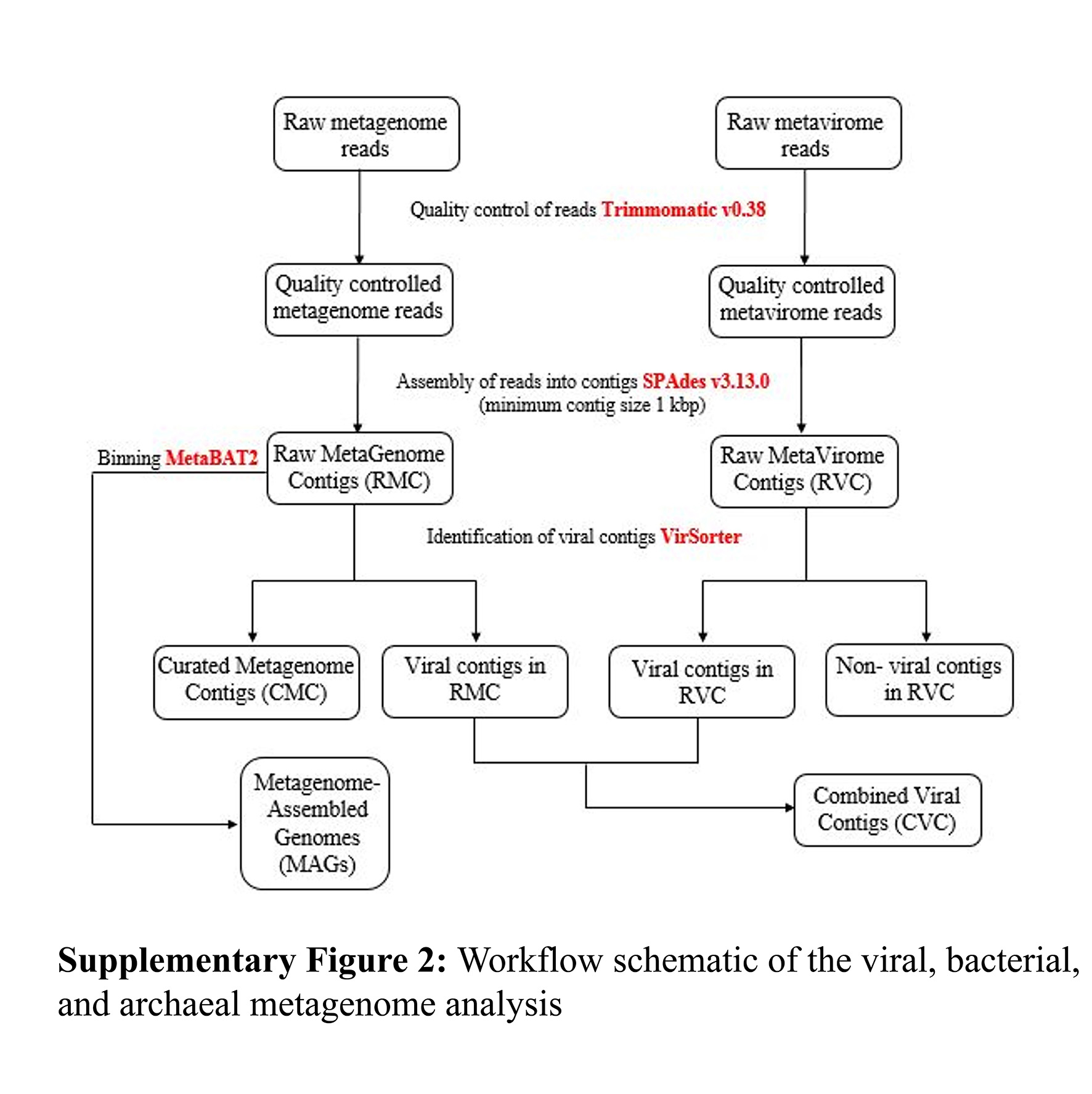

Supplement: Supplementary Figure S2 — Workflow schematic of the viral, bacterial, and archaeal metagenome analysis. [file Image_2.JPEG]

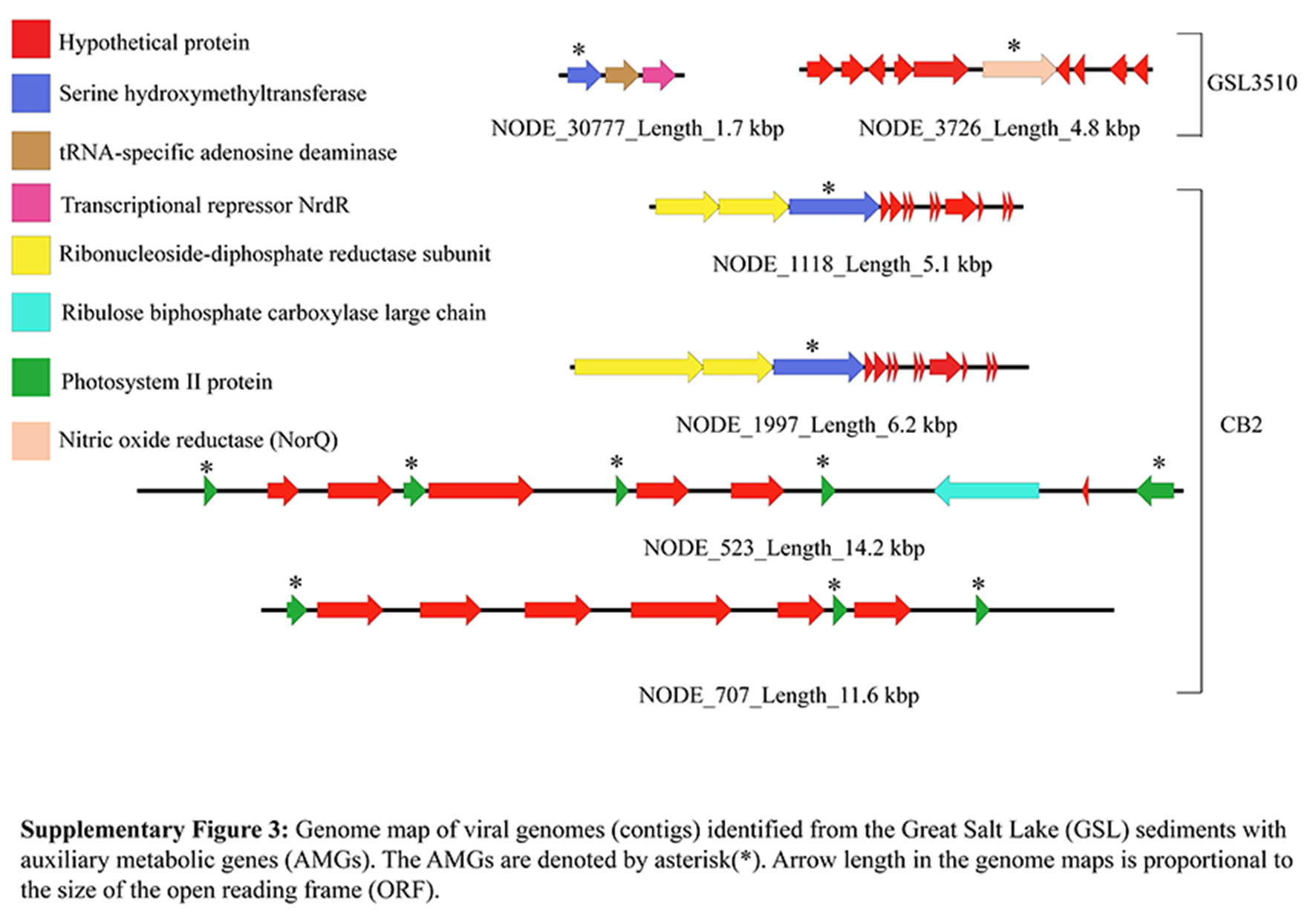

Supplement: Supplementary Figure S3 — Genome map of viral genomes (contigs) identified from the Great Salt Lake sediments with auxiliary metabolic genes (AMGs). The AMGs are denoted by asterisk (*). Arrow length in the genome maps is proportional to the size of the open reading frame (ORF). [file Image_3.TIF]

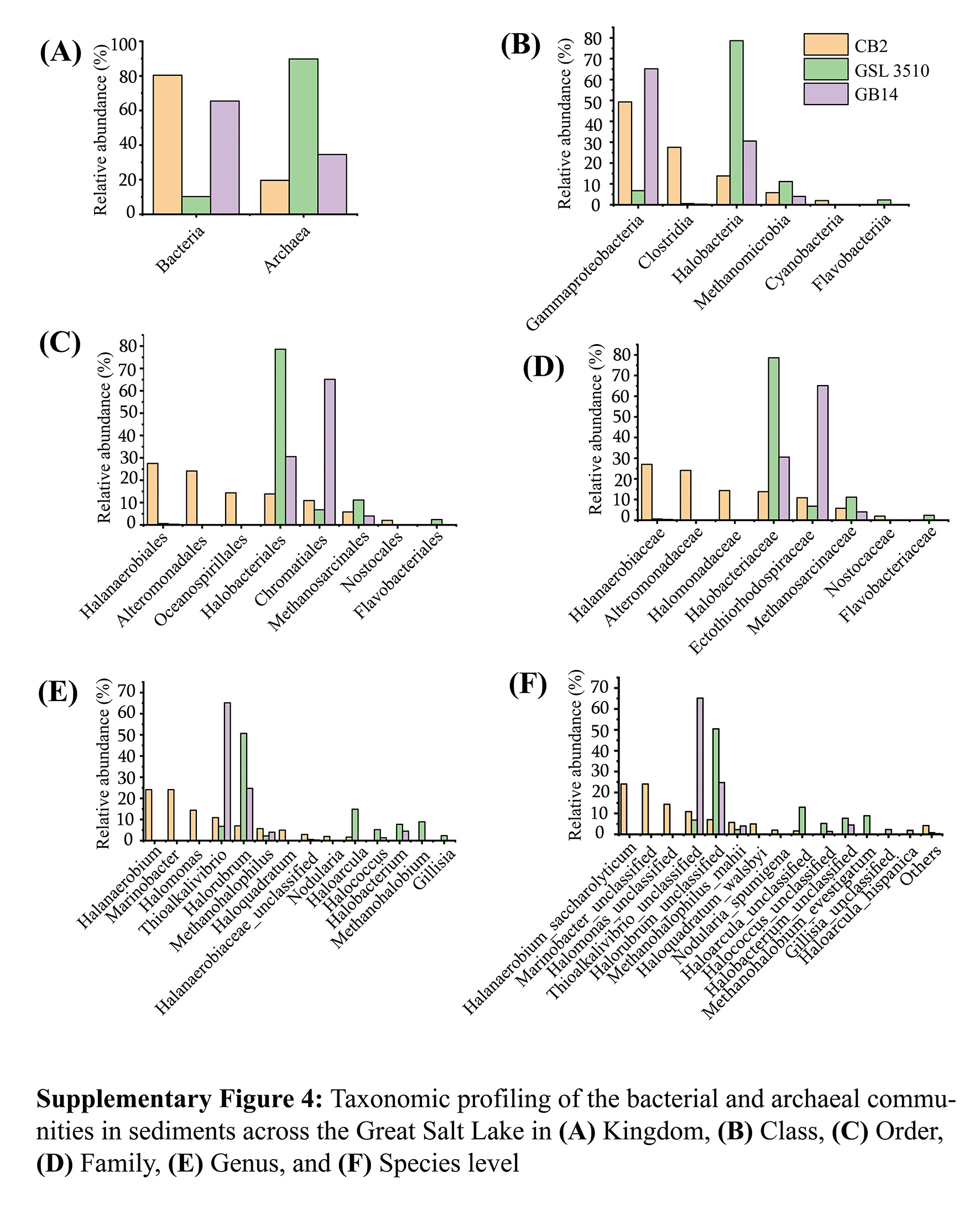

Supplement: Supplementary Figure S4 — Taxonomic profiling of the bacterial and archaeal communities in sediments across the Great Salt Lake in (A) Kingdom, (B) Class, (C) Order, (D) Family, (E) Genus, and (F) Species level. [file Image_4.JPEG]

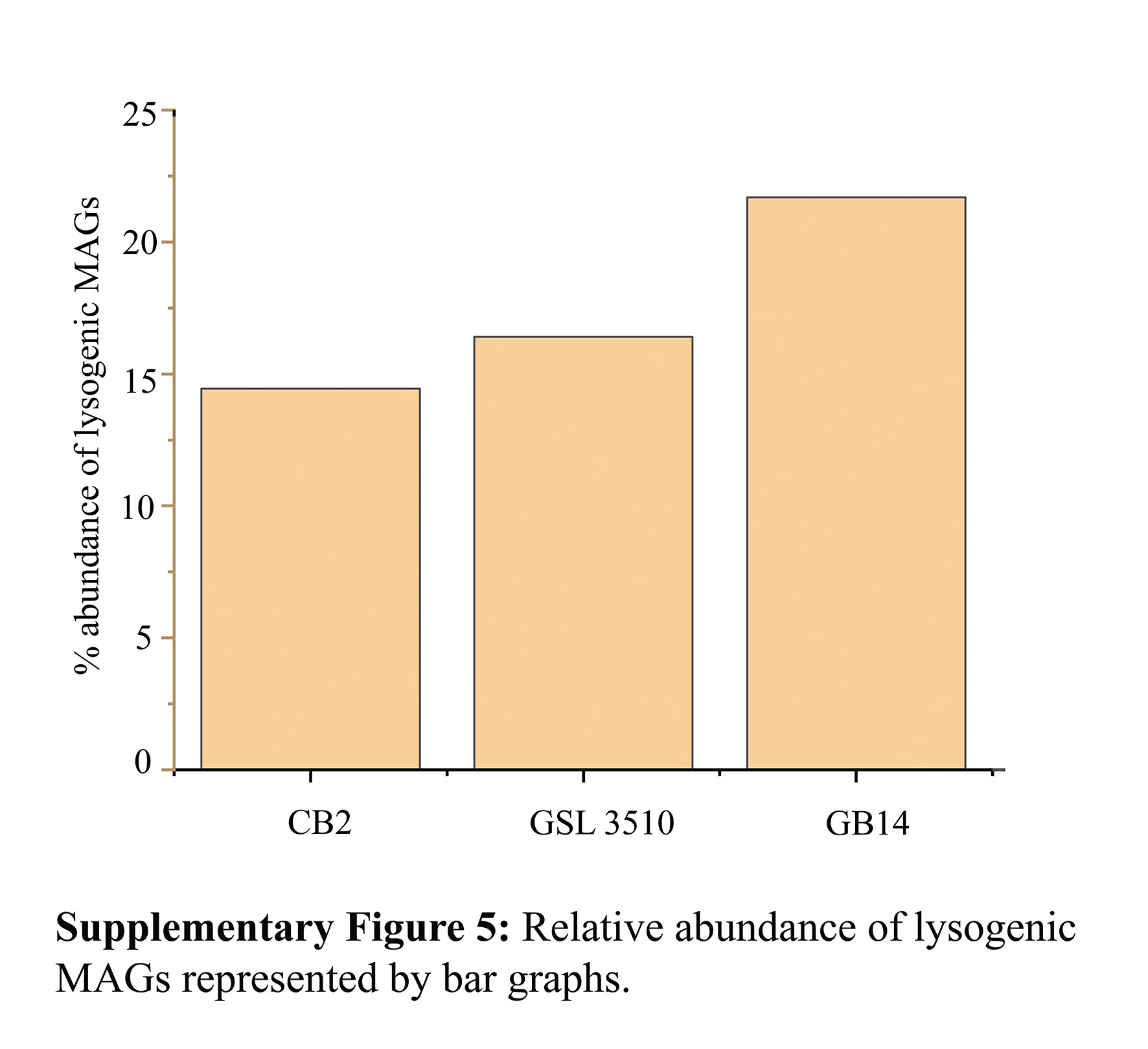

Supplement: Supplementary Figure S5 — Relative abundance of lysogenic MAGs represented by bar graphs. [file Image_5.JPEG]

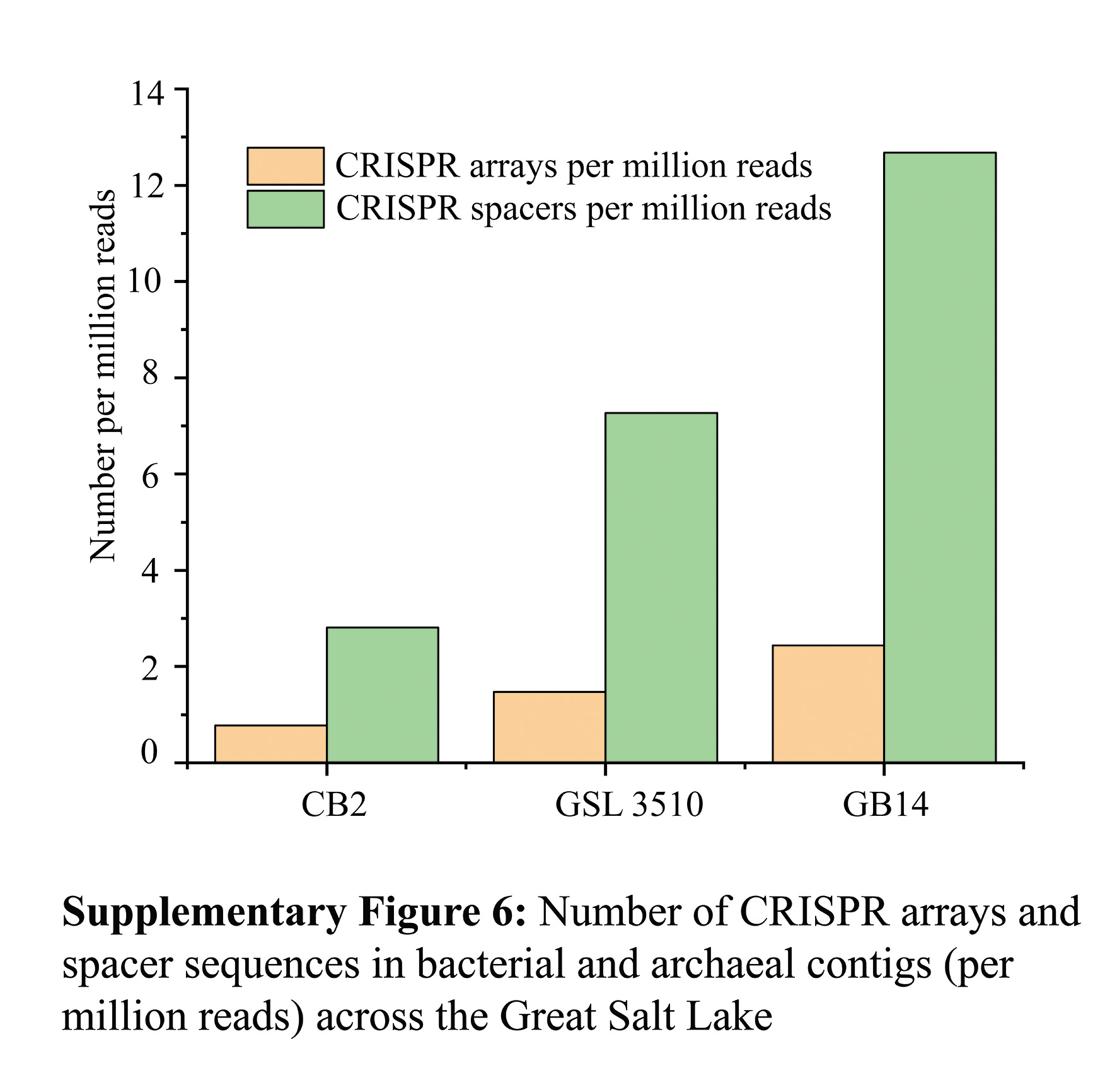

Supplement: Supplementary Figure S6 — Number of CRISPR arrays and spacer sequences in bacterial and archaeal contigs (per million reads) across the Great Salt Lake. [file Image_6.JPEG]

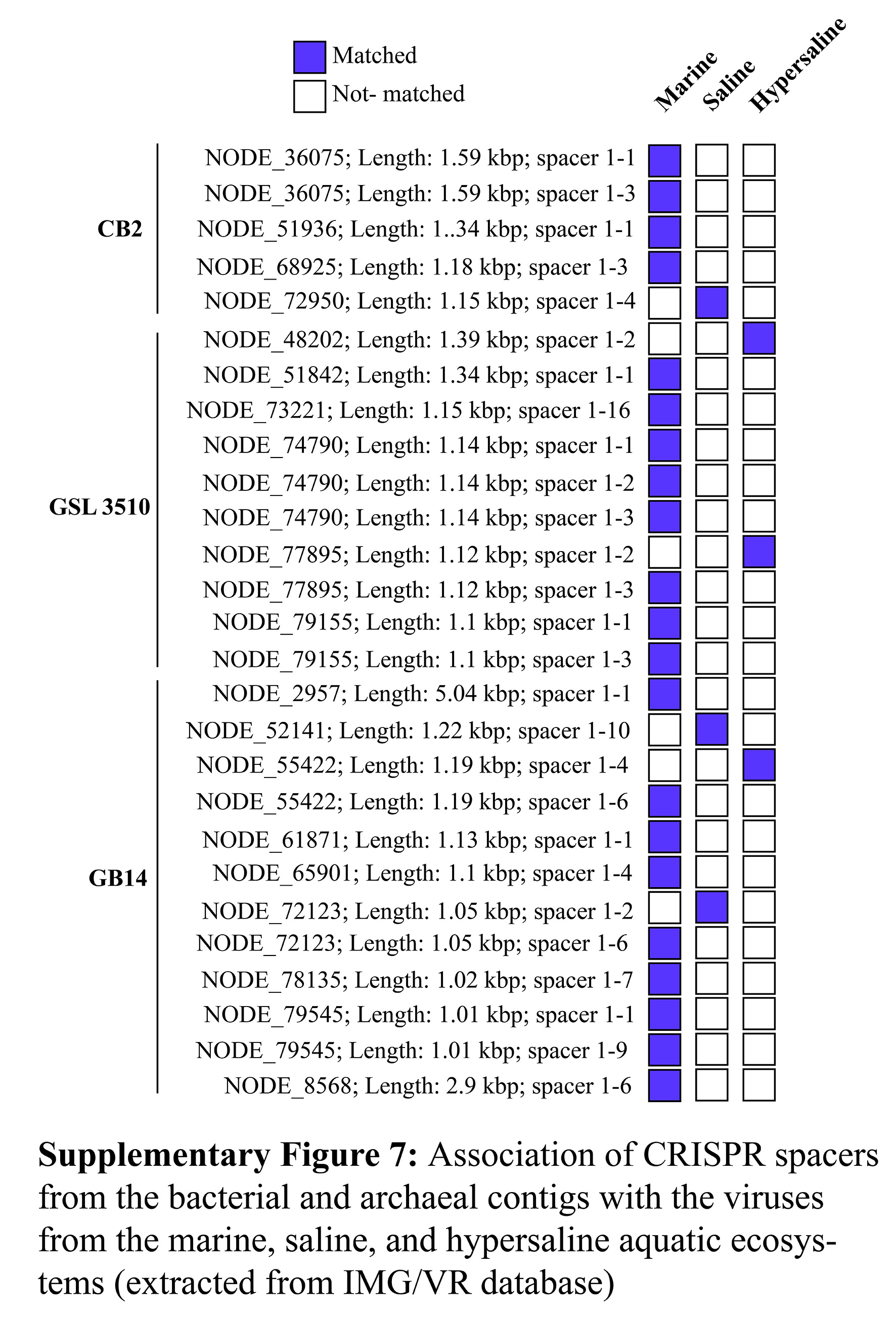

Supplement: Supplementary Figure S7 — Association of CRISPR spacers from the bacterial and archaeal contigs with the viruses from the marine, saline, and hypersaline aquatic ecosystems (extracted from IMG/VR database). [file Image_7.JPEG]

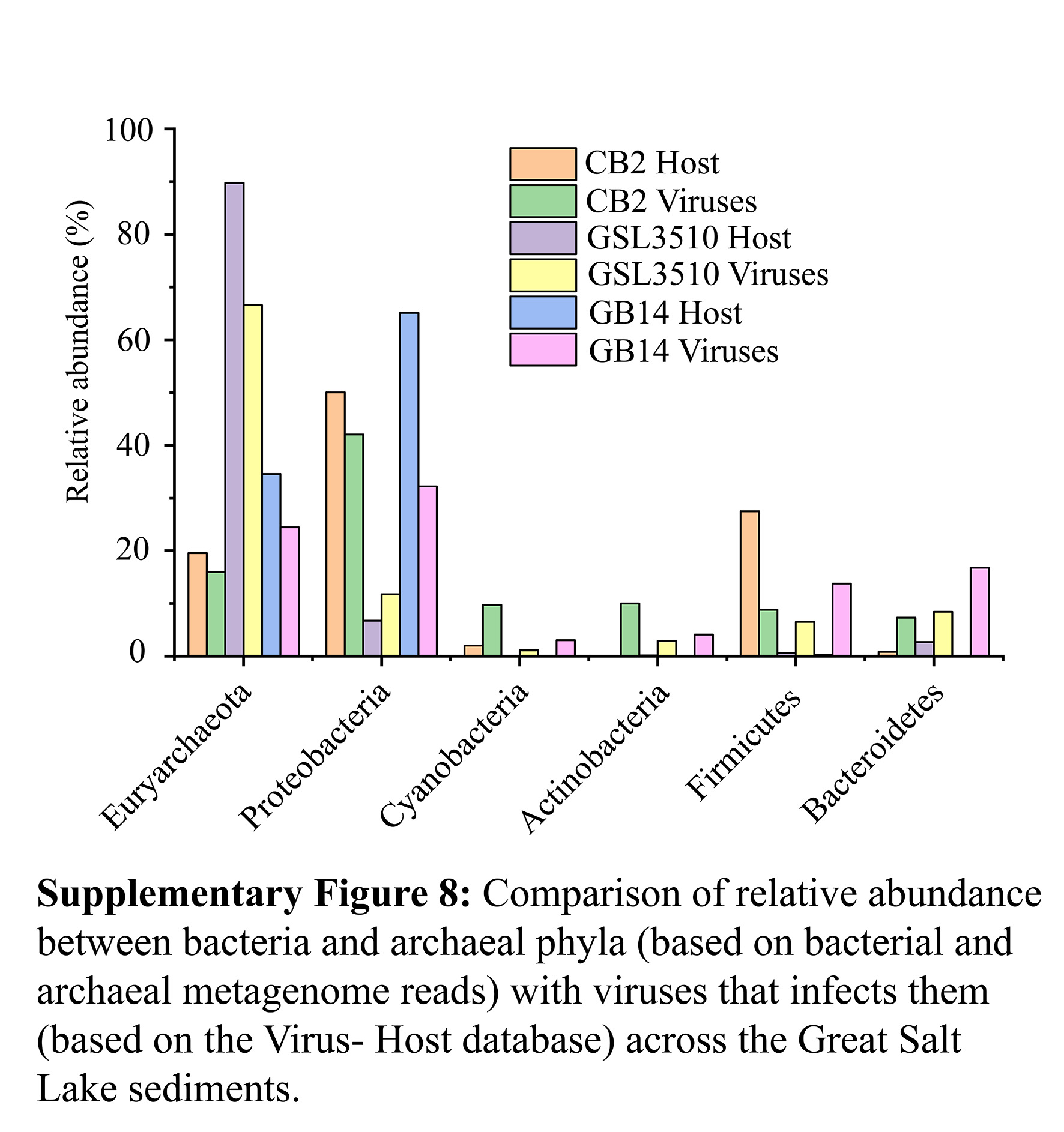

Supplement: Supplementary Figure S8 — Comparison of relative abundance between bacteria and archaeal phyla (based on bacterial and archaeal metagenome reads) with viruses that infects them (based on the Virus–Host database) across the Great Salt Lake sediments. [file Image_8.JPEG]
